# Single-isolate genomic characterization and cgMLST allelic comparison of a bovine mastitis-associated *Staphylococcus aureus* ST151 isolate from Kazakhstan

**DOI:** 10.3389/fvets.2026.1881180

**Published:** 2026-07-30

**Authors:** Vladislava Suchshikh, Ainur Nurpeisova, Kobeikhan Begassyl, Abdilkarim Karimov, Begali Kanatov, Perizat Akshalova, Makay Zheney, Assel Yessenaliyeva, Berik Lessov, Batyrzhan Mustafin, Sairan Dyussenov, Nurkuisa Rametov, Nurbek Aldayarov, Zhuldyz Tlegenova, Malik Yussupov, Raikhan Nissanova, Markhabat Kassenov, Zhandos Abay

**Affiliations:** 1 LLP “Kazakh Scientific Research Veterinary Institute”, Almaty, Kazakhstan; 2 LLP “Tecton Analytics”, Astana, Kazakhstan; 3 Department of Biology, Faculty of Sciences, Kyrgyz-Turkish Manas University, Bishkek, Kyrgyzstan

**Keywords:** antimicrobial resistance, bovine mastitis, cgMLST, Kazakhstan, ST151, *Staphylococcus aureus*, virulence genes, whole-genome sequencing

## Abstract

**Introduction:**

Bovine mastitis caused by *Staphylococcus aureus* remains a major challenge for dairy production, but whole-genome data on mastitis-associated *S. aureus* from Kazakhstan are limited.

**Methods:**

This single-isolate study characterized a milk-derived *S. aureus* isolate obtained from a six-year-old cow with clinically severe, treatment-refractory mastitis in Kostanay Region, Kazakhstan. The isolate was confirmed by bacteriological examination, Gram staining, and species-specific nuc gene PCR. Experimental virulence assessment in mice, histopathological examination of mammary tissue from the same clinical case, Oxford Nanopore MinION whole-genome sequencing, MLST, cgMLST, virulence gene profiling, antimicrobial resistance gene analysis, and supportive disk diffusion screening were performed.

**Results:**

The isolate showed dose-dependent mortality in mice, with an LD50 of approximately 6.3 × 10^6^ CFU. Histopathological examination revealed lesions consistent with acute suppurative mastitis. Whole-genome sequencing and Flye assembly via EPI2ME produced a *de novo* genome assembly of 2,771,674 bp, with 77× mean coverage and a GC content of 32.83%. MLST assigned the isolate to ST151, and cgMLST identified cgST-32654 as the closest profile, with 1,654 of 1,716 loci matched and 62 allelic differences. Virulence profiling detected genes encoding exoenzymes, hemolysins, leukocidins, and egc-associated enterotoxin-like proteins. CARD RGI detected mainly efflux-associated and regulatory antimicrobial resistance-associated loci, whereas ResFinder/PointFinder did not detect acquired antimicrobial resistance genes or known resistance-associated point mutations.

**Discussion:**

Comparative cgMLST minimum-spanning tree analysis indicated allelic relatedness to publicly available bovine-associated ST151 profiles. This genome provides a reference point for future genomic surveillance of mastitis-associated *S. aureus* in Kazakhstan and Central Asia, but population-level conclusions require larger isolate collections.

## 1 Introduction

Bovine mastitis remains one of the most important infectious diseases affecting dairy cattle worldwide, causing substantial economic losses through reduced milk yield, discarded milk, treatment costs, premature culling, and impaired animal welfare (1). In addition to its economic impact, mastitis has public health relevance because milk and dairy production environments may serve as reservoirs for antimicrobial-resistant and potentially zoonotic bacteria. Among the diverse bacterial agents associated with bovine mastitis, *Staphylococcus aureus (S. aureus)* is one of the most clinically important pathogens due to its ability to establish persistent intramammary infections, evade host immune responses, and cause both clinical and subclinical disease (2, 3).

The pathogenicity of *S. aureus* is mediated by a broad repertoire of virulence factors, including adhesins, toxins, enzymes, immune evasion proteins, and biofilm-associated determinants (4). These factors facilitate colonization of mammary tissue, persistence within the udder, and recurrent infection despite treatment. Biofilm formation is particularly important in chronic and subclinical mastitis, as it can reduce antimicrobial penetration, promote bacterial persistence, and contribute to treatment failure and recurrence (5, 6). This phenomenon is especially relevant in dairy herds, where subclinical infections may remain undetected while contributing to reduced milk quality, bacterial dissemination, and economic losses (7, 8). Therefore, understanding the molecular basis of *S. aureus* pathogenicity is essential for improving mastitis control strategies.

Antimicrobial resistance further complicates the management of *S. aureus* mastitis. The emergence of methicillin-resistant *S. aureus* and multidrug-resistant strains in livestock has raised concerns regarding therapeutic failure, persistence of infection, and possible transmission between animals, humans, and the farm environment (9, 10). Livestock-associated lineages, including ST398, have been reported in pigs, humans, and dairy cattle, highlighting the capacity of *S. aureus* to circulate across host species and production systems (11, 12). In mastitic ruminants, *S. aureus* populations show considerable genetic diversity across regions and host species, emphasizing the need for genomic surveillance to clarify lineage distribution, antimicrobial resistance mechanisms, and virulence profiles (13).

Whole-genome sequencing has become a powerful tool for high-resolution characterization of bacterial pathogens. Compared with conventional typing methods, WGS enables simultaneous species confirmation, multilocus sequence typing, detection of antimicrobial resistance and virulence genes, and comparative phylogenetic analysis. In particular, core genome multilocus sequence typing (cgMLST) provides a standardized framework for comparing closely related isolates and assessing allelic relatedness within publicly available genomic datasets. Such approaches are increasingly important for veterinary epidemiology, especially for pathogens such as *S. aureus*, where host adaptation, antimicrobial resistance, and zoonotic potential require integrated genomic investigation.

Despite the recognized importance of bovine mastitis and the public health relevance of *S. aureus*, genomic data on mastitis-associated *S. aureus* from Kazakhstan remain limited (14). Previous regional studies have mainly focused on prevalence, phenotypic antimicrobial resistance, or basic molecular detection, while WGS-based characterization and comparative cgMLST analysis of bovine mastitis-associated *S. aureus* isolates remain scarce (14, 15). This gap limits understanding of the local molecular epidemiology of *S. aureus* mastitis and its relationship to globally circulating bovine-associated lineages.

Therefore, the present study aimed to perform genomic characterization and global cgMLST comparison of a bovine mastitis-associated *S. aureus* isolate from Kazakhstan. The study combined bacteriological identification, molecular confirmation, whole-genome sequencing, MLST and cgMLST analysis, antimicrobial resistance and virulence gene profiling, and pathological assessment. This integrated approach provides a genomic reference point for a mastitis-associated *S. aureus* isolate from Kazakhstan and contributes to the development of genomic surveillance resources for bovine mastitis in Central Asia (16, 17).

## 2 Materials and methods

### 2.1 Sample collection and isolate selection

Milk samples were collected from nine cows with suspected or clinically apparent mastitis during a field investigation at a commercial dairy farm in Kostanay Region, Kazakhstan. Samples were aseptically obtained from affected mammary quarters and transported to the laboratory for bacteriological examination.

Among the recovered bacterial cultures, one molecularly confirmed *S. aureus* isolate obtained from a six-year-old lactating cow with clinical mastitis was selected for whole-genome sequencing and further phenotypic characterization. This isolate was prioritized because it was associated with a clinically evident mastitis case and exhibited pronounced virulence in an experimental mouse model. The present study therefore focused on this milk-derived mastitis-associated *S. aureus* isolate for integrated genomic, virulence, and antimicrobial resistance analysis.

### 2.2 Bacterial isolation and phenotypic identification

Milk samples were subjected to standard bacteriological examination immediately after delivery to the laboratory. Samples were inoculated onto conventional nutrient media, including meat-peptone agar and meat-peptone broth, as well as onto selective chromogenic media used for the presumptive isolation and identification of *S. aureus*. Plates were incubated aerobically at 37 °C for 18–24 h.

Colonies showing morphology consistent with staphylococci were selected and subcultured to obtain pure cultures. Preliminary identification was based on colony morphology, growth characteristics on selective media, and microscopic examination. Smears were prepared from colonies grown on solid media and stained using the Gram method. Gram-positive cocci arranged in clusters were considered presumptive staphylococci and were subjected to further molecular confirmation.

### 2.3 Molecular confirmation of *Staphylococcus aureus* by *nuc* gene PCR

Presumptive *S. aureus* isolates were confirmed by PCR amplification of the species-specific *nuc* gene. The following primers were used: *nuc*-F, 5′-GCGATTGATGGTGATACGGTT-3′, and *nuc*-R, 5′-AGCCAAGCCTTGACGAACTAAAGC-3′. The expected amplicon size was approximately 270 bp.

PCR amplification was performed under the following cycling conditions: initial denaturation at 95 °C for 5 min; 37 cycles of denaturation at 94 °C for 30 s, annealing at 55 °C for 30 s, and extension at 72 °C for 30 s; followed by a final extension at 72 °C for 5 min. PCR products were separated by agarose gel electrophoresis and visualized under UV illumination. Isolates producing a distinct band of the expected size were considered *nuc*-positive and molecularly confirmed as *S. aureus* (18).

### 2.4 Experimental assessment of virulence in mice

The virulence of the selected *S. aureus* isolate was evaluated using a murine intraperitoneal infection model. All procedures were designed to minimize animal suffering and reduce the number of animals used while maintaining scientific validity.

A total of 50 clinically healthy outbred male white mice weighing 18–20 g were used. Animals were randomly allocated into five experimental groups (*n* = 10 per group) using simple random allocation. Blinding was not applied because mortality was the primary endpoint. Mice were housed under standard vivarium conditions at 21–23 °C and 45–65% relative humidity, with a 12 h light/dark cycle and ad libitum access to feed and water. Animals were acclimatized before the experiment and monitored by trained personnel and a veterinarian.

The S. aureus isolate was cultured on meat-peptone agar at 37 °C for 18 h. Bacterial cells were harvested using sterile physiological saline, and viable cell concentrations were determined by serial dilution and colony-forming unit enumeration. Animals were inoculated intraperitoneally with 0.5 mL of bacterial suspension at the following doses: 1 × 10^9^, 1 × 10^8^, 1 × 10^7^, 1 × 10^6^, and 1 × 10^5^ CFU per animal.

Animals were monitored for 10 days post-infection. Clinical assessments were performed twice daily during the first 72 h and once daily thereafter. Humane endpoints were predefined and included severe lethargy, persistent recumbency, loss of righting reflex, inability to access food or water, and severe respiratory distress. Animals reaching humane endpoint criteria were humanely euthanized. At the end of the observation period, all surviving animals were euthanized by CO_2_ inhalation in accordance with institutional ethical guidelines.

The median lethal dose (LD_50_) and 95% confidence interval were calculated using the Kerber method based on mortality distribution across logarithmically spaced doses (19).

### 2.5 Histopathological examination of mammary gland tissue

Mammary gland tissue samples obtained from cows with infectious mastitis and clinically healthy controls were fixed in 10% neutral buffered formalin. The mastitic mammary gland tissue used for histological analysis was obtained from the same cow from which the sequenced *Staphylococcus aureus* isolate had been recovered. Tissue samples were collected from two affected udder quarters, the left front and left rear quarters. From each affected quarter, 25 histological sections were prepared, resulting in a total of 50 sections analyzed from mastitic tissue. For comparison, healthy mammary gland tissue from the corresponding udder quarters of a clinically healthy cow of similar age was processed in the same manner, with 15 histological sections prepared from each analogous quarter.

After fixation, samples were processed according to standard histological procedures, including dehydration in graded ethanol, clearing, paraffin embedding, and sectioning.

Paraffin sections of approximately 5 μm thickness were prepared using a rotary microtome and stained with hematoxylin and eosin according to conventional protocols. Microscopic examination focused on the alveolar-lobular architecture, integrity of the mammary epithelium, inflammatory cell infiltration, exudate accumulation, microabscess formation, necrotic changes, stromal alterations, and vascular lesions. Histological changes in mastitic tissue were compared with the normal structure of bovine mammary gland tissue.

### 2.6 Genomic DNA extraction, whole-genome sequencing, and read processing

Genomic DNA was extracted from a pure culture of the selected *Staphylococcus aureus* isolate using the innuPREP DNA Mini Kit 2.0 according to the manufacturer’s instructions, with an additional enzymatic lysis step suitable for Gram-positive bacteria. Briefly, bacterial biomass from a pure culture was suspended in the appropriate lysis buffer, and 20 μL of lysozyme solution (10 mg/mL) was added to facilitate disruption of the bacterial cell wall. After enzymatic treatment, DNA extraction was continued according to the kit protocol. DNA concentration and purity were assessed using a NanoDrop spectrophotometer.

Whole-genome sequencing was performed using the Oxford Nanopore MinION platform. Sequencing libraries were prepared using the Ligation Sequencing Kit V14 (SQK-LSK114) and run on a FLO-MIN114 flow cell. Basecalling was performed using the high-accuracy model v5.2.0 at 400 bps. Modified base calling, barcoding, alignment, and adaptive sampling were disabled during the sequencing run.

Raw sequencing reads were processed using the EPI2ME platform. *De novo* genome assembly was performed using Flye via EPI2ME v2.9.6 in April 2026. The assembly was generated as a *de novo* bacterial genome assembly from Oxford Nanopore long-read data. No additional standalone polishing software could be verified from the retained workflow records; therefore, only the confirmed EPI2ME/Flye assembly workflow is reported here. Assembly quality metrics, including total assembly length, number of contigs, N50, largest contig, ambiguous nucleotides, GC content, and sequencing depth, were recorded and used to evaluate assembly quality.

### 2.7 Genome assembly quality assessment, annotation, and deposition

Assembly quality was evaluated using the metrics generated during the EPI2ME/Flye workflow and NCBI genome submission. The final assembly had a total length of 2,771,674 bp, a mean genome coverage of 77×, an N50 of 2,771,674 bp, and a GC content of 32.83%. No ambiguous nucleotides were present in the final assembly. The assembly represented the final *de novo* genome version submitted to NCBI.

Genome annotation was performed using the NCBI Prokaryotic Genome Annotation Pipeline (PGAP) during GenBank submission. Circular genome visualization was performed using Proksee. The circular genome map included coding sequences, GC content, GC skew, and antimicrobial resistance-associated loci identified using the Comprehensive Antibiotic Resistance Database (CARD) (20).

The genome sequence was deposited in NCBI GenBank under WGS accession number JBYFBM000000000. The sequencing project is available under BioProject accession number PRJNA1465568, and the corresponding BioSample accession number is SAMN59747580. The NCBI genome submission number is SUB16182760.

### 2.8 MLST and cgMLST analysis

Multilocus sequence typing was performed in silico using the assembled genome sequence and the *S. aureus* MLST scheme available in the PubMLST database. The allelic profile was determined based on the seven housekeeping loci included in the scheme: *arcC*, *aroE*, *glpF*, *gmk*, *pta*, *tpi*, and *yqiL*. The sequence type was assigned according to the corresponding allelic profile in PubMLST (21).

For comparative cgMLST analysis, public *Staphylococcus aureus* ST151 records were retrieved from the PubMLST database. Records without sufficient cgMLST allelic profile data for minimum-spanning tree analysis were excluded. After filtering, the cleaned dataset included 183 public ST151 isolates and the Kazakhstan query isolate SA_KSRVI_2025_1. Available metadata, including isolate identifier, isolate name, country, year, disease status, and source, were summarized in Supplementary Table S1. Missing metadata were reported as “not available” and were not inferred. Allelic relatedness was visualized using GrapeTree with the MSTreeV2 algorithm (41).

Core genome multilocus sequence typing was performed using the PubMLST *S. aureus* cgMLST scheme comprising 1,716 loci. The assembled genome was queried against the cgMLST database to identify the closest cgMLST profile, determine the number of matched loci, and estimate allelic differences.

### 2.9 Virulence gene analysis

Virulence-associated genes were identified using VirulenceFinder and curated virulence factor databases. Detected genes were grouped according to their predicted functional categories, including exoenzymes, hemolysins, leukocidins, egc-associated enterotoxin-like genes, immune evasion-associated genes, adhesion-related genes, and biofilm-associated determinants.

### 2.10 Antimicrobial resistance gene analysis

Antimicrobial resistance-associated loci were analyzed using CARD RGI, ResFinder, and PointFinder (20). CARD RGI was used to identify AMR-associated genes, regulatory loci, efflux-associated loci, and target-alteration-related markers. ResFinder was used to screen for acquired antimicrobial resistance genes, and PointFinder was used to identify known resistance-associated chromosomal point mutations in *S. aureus*. CARD RGI hits were interpreted together with ResFinder/PointFinder results and supportive phenotypic disk diffusion screening.

### 2.11 Phenotypic antimicrobial susceptibility testing

Phenotypic antimicrobial susceptibility screening of the sequenced *S. aureus* isolate was performed using the disk diffusion method. A bacterial suspension was prepared from a fresh pure culture and evenly inoculated onto meat-peptone agar plates. Antibiotic disks were then placed on the agar surface.

The tested antibiotics included penicillin, ampicillin, amoxicillin/clavulanic acid, piperacillin, tetracycline, minocycline, levofloxacin, ciprofloxacin, and oxacillin. Oxacillin was tested using an OX-1 disk containing 1 μg of oxacillin as part of the supportive phenotypic antimicrobial susceptibility screening panel. Plates were incubated aerobically at 35–37 °C for 18–24 h. After incubation, inhibition zone diameters were measured in millimeters. Because the testing was performed on meat-peptone agar rather than a standardized Mueller–Hinton agar system, the results are reported as inhibition zone diameters and used as supportive phenotypic screening data rather than formal categorical antimicrobial susceptibility interpretation.

## 3 Results

### 3.1 Clinical origin, sample flow, and bacteriological identification

A total of nine milk samples were collected from cows with suspected or clinically apparent mastitis on a commercial dairy farm in Kostanay Region, Kazakhstan. One *S. aureus* isolate recovered from milk of a six-year-old lactating cow with clinically severe mastitis was selected for detailed phenotypic and genomic characterization.

To clarify the selection of the sequenced isolate, a sample-flow table was added summarizing all nine milk samples, bacteriological and PCR results, identified microorganisms, and the rationale for selecting one isolate for whole-genome sequencing. All nine milk samples were systematically analyzed using bacteriological and molecular approaches. Most samples demonstrated polymicrobial infections involving *Escherichia coli*, Streptococcus spp., and Proteus spp., whereas only sample No. 5 yielded a pure culture of *Staphylococcus aureus*. This isolate was selected for WGS because it originated from a severe treatment-refractory clinical mastitis case and was recovered as a pure *S. aureus* culture (see Table 1).

**Table 1:** Sample flow and microbiological findings.

Sample no.	Animal, age	Bacteriological culture result	PCR result	Identified microorganisms	Selected for sequencing
1	Cow, 8 years	Positive culture	Positive	*Escherichia coli*, Streptococcus spp.	No
2	Cow, 6 years	Positive culture	Positive	*Escherichia coli*	No
3	Cow, 7 years	Positive culture	Positive	Streptococcus spp., Proteus spp.	No
4	Cow, 4 years	Positive culture	Positive	*Escherichia coli*	No
5	Cow, 6 years	Positive culture	Positive	*Staphylococcus aureus*	Yes
6	Cow, 6 years	Positive culture	Positive	*Escherichia coli*	No
7	Cow, 9 years	Positive culture	Positive	Streptococcus spp., Proteus spp.	No
8	Cow, 5 years	Positive culture	Positive	Proteus spp.	No
9	Cow, 6 years	Positive culture	Positive	*Escherichia coli*	No

Clinical examination of the affected cow revealed involvement of multiple mammary quarters, firm udder consistency on palpation, and purulent material in the milk, consistent with purulent-catarrhal mastitis. According to the farm veterinarian, the animal showed poor clinical response to antimicrobial treatment used on the farm and was subsequently scheduled for culling. The isolate was prioritized for further investigation because it originated from a severe mastitis case and demonstrated pronounced pathogenicity in the experimental mouse model.

Bacteriological examination revealed colonies with morphology consistent with staphylococci on selective chromogenic medium and conventional nutrient media. Growth was also observed on meat-peptone agar and in meat-peptone broth. Microscopic examination of Gram-stained smears prepared from colonies grown on solid medium showed Gram-positive cocci arranged predominantly in clusters, supporting presumptive identification as staphylococci (see Figure 1).

**Figure 1:**
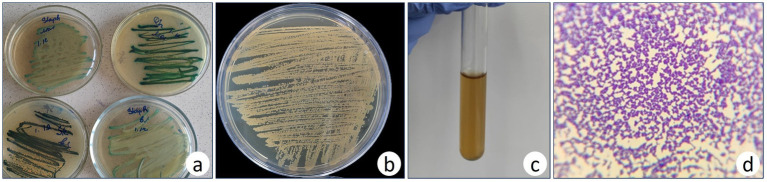
Culture and microscopic identification of *S. aureus*. **(a)** Growth on selective chromogenic medium. **(b)** Growth on meat-peptone agar. **(c)** Growth in meat-peptone broth. **(d)** Gram-stained smear of the culture grown on solid medium showing Gram-positive cocci arranged in clusters.

### 3.2 Molecular confirmation by *nuc* gene PCR

PCR amplification of the species-specific *nuc* gene confirmed the molecular identity of the presumptive *S. aureus* isolate. Agarose gel electrophoresis showed a distinct amplification product of the expected size of approximately 270 bp. The selected isolate was therefore confirmed as *nuc*-positive *S. aureus* and was used for subsequent whole-genome sequencing and genomic characterization (see Figure 2).

**Figure 2:**
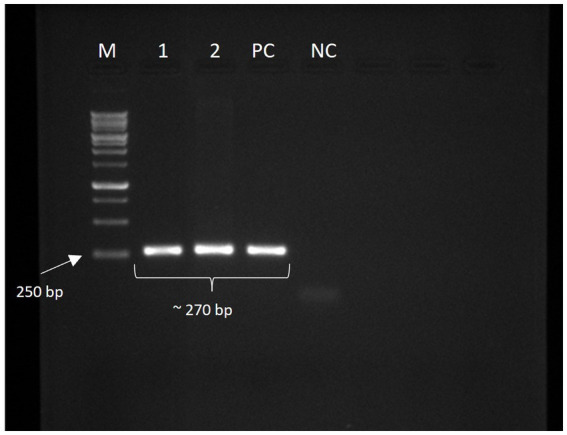
PCR confirmation of *S. aureus* by amplification of the species-specific *nuc* gene. Lane M: DNA ladder; lanes 1 and 2: duplicate PCR reactions of the nuc-positive isolate showing an amplicon of approximately 270 bp; lane PC: positive control (*S. aureus* reference/collection strain); lane NC: negative control (H_2_O).

### 3.3 Experimental virulence assessment in mice

The pathogenicity of the selected *S. aureus* isolate was assessed in white outbred mice following intraperitoneal inoculation with serial ten-fold dilutions of the bacterial suspension. Animals were monitored for 10 days after inoculation. Mortality was dose-dependent, and bacteriological examination of dead animals confirmed recovery of the inoculated *S. aureus* culture from pathological material.

Based on mortality data, the median lethal dose calculated by the Kerber method was approximately 6.3 × 10^6^ CFU, indicating marked virulence of the isolate under the experimental conditions.

### 3.4 Histopathological features of infectious mastitis

Histopathological examination was performed on mammary gland tissue obtained from the same cow from which the sequenced *S. aureus* isolate was recovered. The examined tissue originated from two affected udder quarters, thereby directly linking the pathological findings to the clinical case from which the genome-sequenced isolate was obtained.

Histological examination of mammary gland tissue from clinically healthy cows revealed a typical alveolar-lobular organization of the bovine mammary gland (Figure 3A). Secretory alveoli were lined by simple cuboidal to low columnar epithelium, and the glandular parenchyma was supported by loose connective tissue stroma containing blood vessels, lymphatic structures, and scattered resident immune cells. No inflammatory, degenerative, necrotic, or atypical cellular changes were observed in the normal tissue.

**Figure 3:**
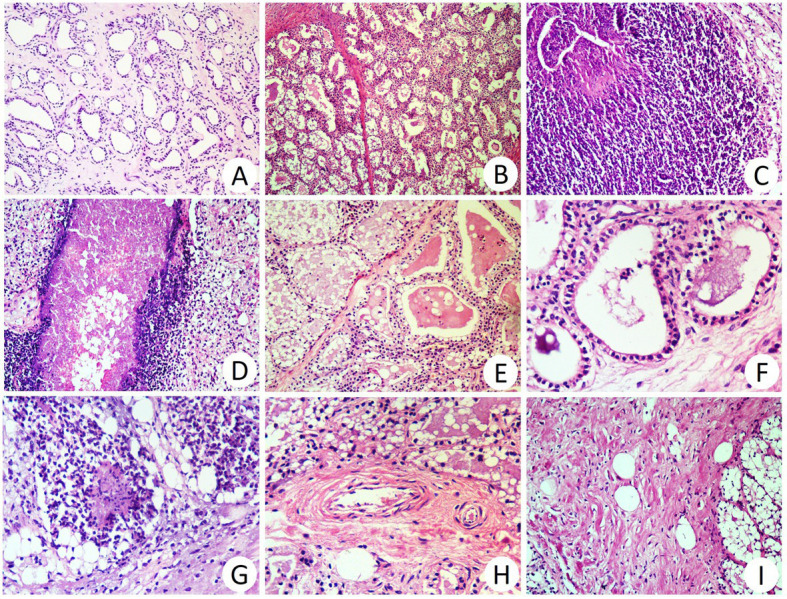
Histopathological structure of the bovine mammary gland under normal conditions and during infectious mastitis. **(A)** Normal mammary gland showing typical alveolar-lobular organization. **(B–D)** Marked inflammatory and destructive changes with prominent neutrophilic infiltration and disruption of the normal alveolar-lobular architecture. **(E,F)** Degenerative changes of the epithelial lining, including cellular dystrophy, desquamation, and necrosis. **(C,G)** Microabscess formation characterized by focal accumulations of neutrophils surrounding necrotic tissue debris. **(H)** Vascular alterations, including endothelial swelling, vascular dilation, and focal disruption of vascular walls. **(I)** Stromal alterations, including edema, loosening, focal necrotic changes, and disorganization of the fibrous connective tissue framework. Hematoxylin and eosin staining. Magnification: ×200 **(A–E,I)** and ×400 **(F–H)**.

In contrast, mammary gland tissue affected by infectious mastitis showed severe inflammatory and destructive alterations with marked disruption of the normal alveolar-lobular architecture (Figures 3B–D). In many examined areas, the secretory epithelial lining showed pronounced degenerative changes, including cellular dystrophy, desquamation, and necrosis (Figures 3E,F). Myoepithelial cells were markedly reduced or absent in affected regions. The lumina of numerous alveoli were narrowed or completely obliterated due to edema of the alveolar walls and accumulation of inflammatory exudate.

The affected alveolar lumina contained abundant seropurulent to fibrinopurulent exudate composed mainly of cellular debris and numerous neutrophils, with occasional macrophages and lymphocytes. Multifocal microabscesses were observed as localized accumulations of neutrophils surrounding necrotic tissue remnants (Figures 3C,G). Multifocal areas of coagulative and liquefactive necrosis were also present, indicating extensive tissue damage within the inflamed mammary parenchyma.

The lactiferous ducts were also involved in the inflammatory process and showed epithelial destruction, luminal obstruction, and accumulation of purulent exudate. Blood vessels displayed marked pathological changes, including endothelial swelling, vascular dilation, and focal disruption of vascular walls (Figure 3H). The interalveolar connective tissue was severely edematous and densely infiltrated with inflammatory cells, predominantly neutrophils, accompanied by stromal loosening, focal necrotic changes, and disorganization of the fibrous stromal framework (Figure 3I).

Overall, the observed histopathological features, including massive neutrophilic infiltration, microabscess formation, fibrinopurulent exudation, epithelial destruction, necrotic changes, stromal edema, and vascular alterations, were consistent with acute suppurative mastitis compatible with staphylococcal etiology.

### 3.5 Whole-genome sequencing and genome assembly

The selected *S. aureus* isolate was subjected to whole-genome sequencing using Oxford Nanopore Technologies. *De novo* assembly generated a highly contiguous genome consisting of a single circular chromosomal sequence. The total assembly length was 2,771,674 bp, with an N50 of 2,771,674 bp and a largest fragment of 2,771,674 bp. No scaffolds or ambiguous nucleotides were present in the final assembly. The mean sequencing coverage was 77×, and the GC content was 32.83%, consistent with the expected genomic characteristics of *S. aureus*.

The circular genome map demonstrated the distribution of coding sequences, GC content, GC skew, and antimicrobial resistance-associated loci across the chromosome (Figure 4).

**Figure 4:**
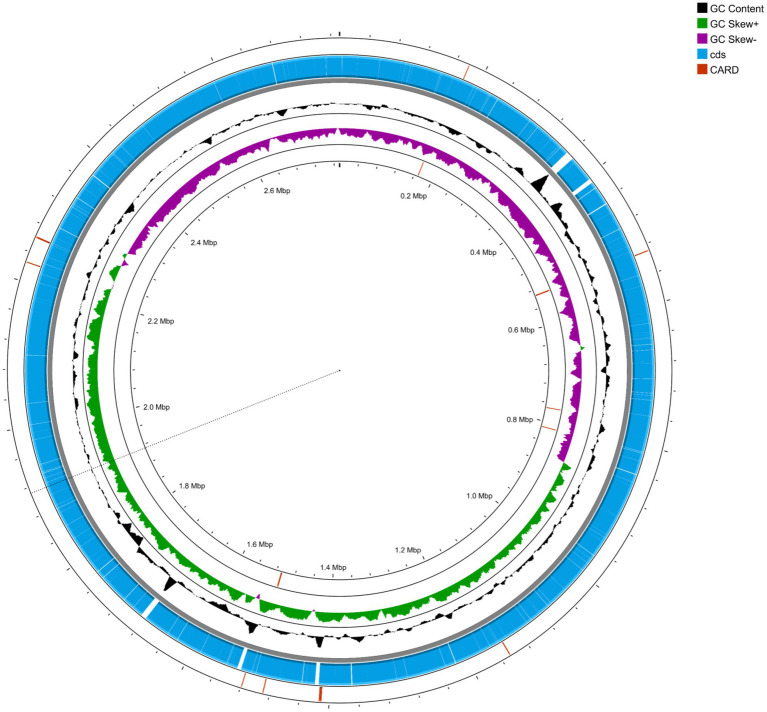
Circular genome map of the mastitis-associated *S. aureus* isolate generated using Proksee. The map shows the organization of the single circular chromosome of the isolate. The coding sequences (CDS) are shown in the outer blue ring, while internal tracks represent GC content and GC skew. CARD hits corresponding to antimicrobial resistance-associated loci are indicated by red/orange markers. Genome coordinates are shown in megabase pairs (Mbp).

### 3.6 MLST and cgMLST characterization

In silico multilocus sequence typing assigned the isolate to sequence type ST151. The allelic profile consisted of *arcC*-6, *aroE*-72, *glpF*-12, *gmk*-43, *pta*-49, *tpi*-67, and *yqiL*-59 (see Table 2).

**Table 2:** MLST allelic profile of the mastitis-associated *S. aureus* isolate.

Locus	Allele
*arcC*	6
*aroE*	72
*glpF*	12
*gmk*	43
*pta*	49
*tpi*	67
*yqiL*	59
Sequence type	ST151

Core genome MLST analysis assigned the isolate to the closest cgMLST profile cgST-32654. A total of 1,654 of 1,716 loci were matched, corresponding to 96.4% locus identity, with 62 allelic differences from the closest cgMLST profile. These results placed the Kazakhstan isolate within the ST151 lineage and supported its comparison with publicly available bovine-associated ST151 isolates.

### 3.7 Virulence-associated gene profile

Virulence gene analysis revealed several virulence-associated loci in the mastitis-associated *Staphylococcus aureus* isolate. The detected genes included exoenzyme-associated genes aur, encoding aureolysin, and splA and splB, encoding serine proteases. Genes encoding gamma-hemolysin components, including hlgA, hlgB, and hlgC, were also identified. In addition, leukocidin-associated genes lukD and lukE were present.

The isolate also carried the genes seg, sei, sem, sen, seo, and seu. These genes were interpreted as egc-associated enterotoxin-like genes rather than classical enterotoxin genes. Their detection indicates the presence of virulence-associated genomic loci, but does not by itself demonstrate toxin expression, toxin production, or food poisoning risk. Therefore, their biological significance should be interpreted cautiously and requires further experimental confirmation.

Overall, the detected virulence-associated gene profile suggests that the isolate carries genomic determinants potentially involved in tissue damage, inflammatory response, immune cell targeting, and host-pathogen interaction. However, the presence of these genes was interpreted as genomic evidence of virulence-associated potential rather than direct proof of gene expression or toxin activity. Detailed VirulenceFinder gene-level results, including identity, query/template length, genomic position, predicted function, and accession number, are provided in Supplementary Table S2 (see Table 3).

**Table 3:** Virulence-associated genes detected in the mastitis-associated *S. aureus* isolate.

Functional category	Detected genes	Putative role
Exoenzymes	*aur*, *splA*, *splB*	Protein degradation, tissue invasion, host-pathogen interaction
Hemolysins	*hlgA*, *hlgB*, *hlgC*	Cell membrane damage and inflammation- associated processes
Leukocidins	*lukD*, *lukE*	Leukocyte targeting and immune interaction
egc-associated enterotoxin-like genes	*seg*, *sei*, *sem*, *sen*, *seo*, *seu*	Virulence-associated loci; toxin expression or production was not assessed
Host immune modulation	Not detected	Not identified in the applied analysis

### 3.8 Antimicrobial resistance-associated genes

Analysis of antimicrobial resistance-associated loci was performed using CARD RGI and ResFinder/PointFinder. CARD RGI identified several AMR-associated loci, mainly efflux-associated and regulatory genes, including norA, norC, sdrM, sepA, LmrS, mgrA, mepR, kdpD, arlR, and target-alteration-related markers. These loci were interpreted cautiously because many of them are chromosomal, intrinsic, regulatory, or efflux-associated genes and should not be interpreted alone as evidence of categorical antimicrobial resistance or multidrug resistance.

In contrast, ResFinder did not detect acquired antimicrobial resistance genes in the genome, and PointFinder did not identify known resistance-associated chromosomal point mutations in the analyzed *Staphylococcus aureus* targets. Therefore, the isolate was not classified as MRSA, and no mecA-, SCCmec-, or mec-complex-based interpretation was made.

Overall, the antimicrobial resistance analysis did not provide evidence for acquired antimicrobial resistance genes or known resistance-associated point mutations. The CARD RGI findings were interpreted as AMR-associated efflux or regulatory loci only. Detailed CARD RGI gene-level results are provided in Supplementary Table S3. ResFinder did not detect acquired antimicrobial resistance genes, and PointFinder did not identify known resistance-associated point mutations; therefore, CARD RGI efflux-associated and regulatory loci were interpreted cautiously and not as standalone evidence of categorical antimicrobial resistance (see Table 4).

**Table 4:** Antimicrobial resistance-associated loci detected in the mastitis-associated *Staphylococcus aureus* isolate and cautious interpretation based on CARD RGI, ResFinder, and PointFinder analyses.

Functional category	Detected loci/finding	Interpretation
Efflux-associated loci	*norA*, *norC*, *sdrM*, *sepA*, *LmrS*	CARD RGI-detected efflux-associated loci; not interpreted alone as evidence of categorical antimicrobial resistance or multidrug resistance
Efflux-regulatory or regulatory loci	*mgrA*, *mepR*, *kdpD*, *arlR*	Regulatory or efflux-modulating loci; biological impact requires phenotypic or functional confirmation
Fosfomycin target alteration-associated markers	*glpT*, *murA*	CARD RGI variant-model hits; interpreted cautiously because ResFinder/PointFinder did not predict fosfomycin resistance
Low-confidence glycopeptide-associated hit	*vanT*-like hit	Low-identity/partial hit; not interpreted as evidence of vancomycin resistance
Acquired AMR genes and known resistance-associated point mutations	Not detected by ResFinder/PointFinder	No acquired AMR genes or known resistance-associated point mutations were detected using ResFinder/PointFinder; no MRSA, SCCmec, or mec-complex interpretation was made

### 3.9 Phenotypic antimicrobial susceptibility testing

Phenotypic antimicrobial susceptibility screening was performed to complement the genomic analysis of antimicrobial resistance-associated loci. The inhibition zone diameters measured by disk diffusion are presented in Table 5. The isolate showed inhibition zone diameters of 32–35 mm for penicillin, 20 mm for ampicillin, 30 mm for amoxicillin/clavulanic acid, 35 mm for piperacillin, 25 mm for tetracycline, 25 mm for minocycline, 25–26 mm for levofloxacin, and 26–27 mm for ciprofloxacin. Oxacillin testing using an OX-1 disk containing 1 μg of oxacillin produced an inhibition zone diameter of 12 mm.

**Table 5:** Phenotypic antimicrobial susceptibility screening of the sequenced *Staphylococcus aureus* isolate by disk diffusion.

Antibiotic	Disk code	Disk content	Inhibition zone diameter, mm
Penicillin	P	10 units	32–35
Ampicillin	AM	20 μg	20
Amoxicillin/clavulanic acid	AMC	30 μg	30
Piperacillin	PRL	100 μg	35
Tetracycline	TE	30 μg	25
Minocycline	MH	30 μg	25
Levofloxacin	LEV	5 μg	25–26
Ciprofloxacin	CIP	5 μg	26–27
Oxacillin	OX-1	1 μg	12

Disk diffusion testing was performed on meat-peptone agar; therefore, the results are reported as inhibition zone diameters and used as supportive phenotypic screening data rather than formal categorical susceptibility interpretation.

The small inhibition zone around oxacillin provided supportive screening evidence compatible with reduced oxacillin susceptibility. However, because the assay was performed on meat-peptone agar rather than a standardized Mueller-Hinton agar system, the results are presented as supportive disk diffusion screening data rather than formal categorical antimicrobial susceptibility interpretation or formal confirmation of MRSA status.

Representative disk diffusion plates are provided in Supplementary Figure S1.

### 3.10 Comparative cgMLST analysis and allelic relatedness

To evaluate the allelic relatedness of the Kazakhstan isolate within the ST151 lineage, cgMLST-based comparative analysis was performed using publicly available *S. aureus* ST151 profiles from the PubMLST database. The resulting cgMLST minimum-spanning tree showed that ST151 profiles were represented by isolates from multiple geographic regions, including Europe, North America, and Oceania. Available metadata indicated that many ST151 profiles were associated with animal or milk sources, including mastitis-related samples.

The Kazakhstan isolate was positioned within the ST151 cgMLST framework and was highlighted as the query isolate from the present study. The closest cgMLST profile was cgST-32654, consistent with the MLST assignment and supporting allelic relatedness to publicly available bovine-associated ST151 profiles. The majority of closely related isolates with available metadata originated from countries such as Ireland, the United Kingdom, the Netherlands, the USA, Canada, Denmark, Switzerland, Australia, and New Zealand. Because this analysis was based on cgMLST allelic profiles and visualized using a minimum-spanning tree, the results are interpreted as allelic relatedness rather than formal phylogenetic inference (see Figure 5).

**Figure 5:**
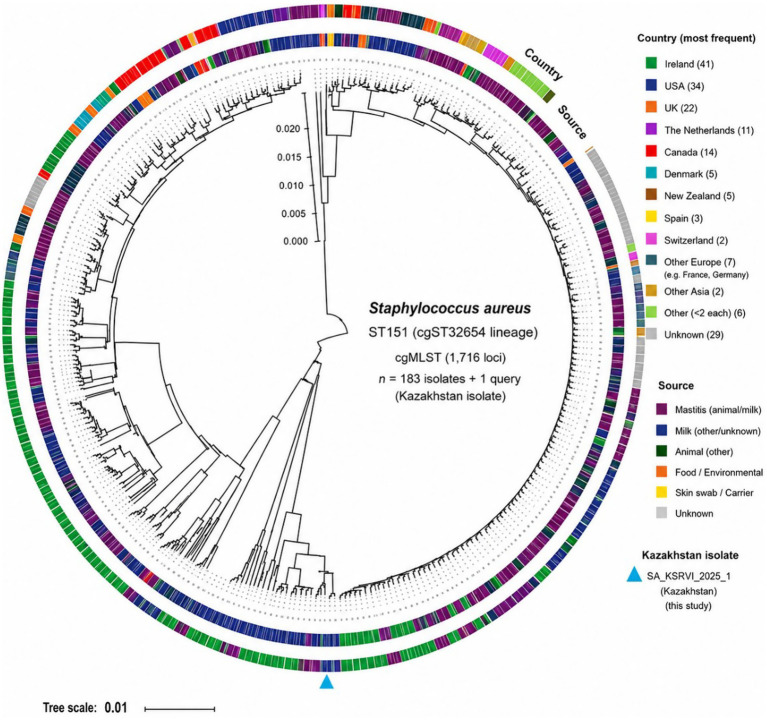
cgMLST minimum-spanning tree of *Staphylococcus aureus* ST151 lineage isolates based on 1,716 core genome loci. The cleaned public ST151 dataset used to generate this cgMLST minimum-spanning tree is summarized in Supplementary Table S2.

The allelic relationships among *S. aureus* ST151 isolates were inferred using cgMLST analysis and visualized as a circular minimum-spanning tree. Publicly available ST151 profiles from the PubMLST database were included for comparison with the Kazakhstan isolate obtained in this study. The Kazakhstan isolate is indicated by a blue triangle. Colored outer rings represent isolate metadata, including country of origin and source category. Branch lengths correspond to allelic differences among cgMLST profiles. cgMLST profiling and visualization were performed using the PubMLST *S. aureus* database and GrapeTree with the MSTreeV2 algorithm. The output was interpreted as cgMLST allelic relatedness rather than formal SNP-based phylogenetic inference.

## 4 Discussion

This study provides an integrated genomic and phenotypic characterization of a bovine mastitis-associated *S. aureus* isolate from Kazakhstan. The isolate was confirmed as *S. aureus* by conventional bacteriological and molecular approaches and further characterized using whole-genome sequencing, MLST, cgMLST, antimicrobial resistance gene detection, virulence gene profiling, experimental pathogenicity assessment, and histopathology. Together, these data provide a high-resolution view of the genetic and pathogenic features of a mastitis-associated *S. aureus* isolate from a region where genomic information on this pathogen remains limited (14, 15).

A key finding of this study was the assignment of the Kazakhstan isolate to sequence type ST151 based on WGS-derived MLST. The allelic profile corresponded to *arcC*-6, *aroE*-72, *glpF*-12, *gmk*-43, *pta*-49, *tpi*-67, and *yqiL*-59. ST151 is widely recognized as a bovine-associated lineage of *S. aureus* and has been reported in dairy cattle and mastitis-associated isolates in several geographic regions (11, 22, 23). The identification of ST151 in a bovine mastitis-associated isolate from Kazakhstan is therefore epidemiologically relevant, as it links the regional isolate to a globally distributed lineage adapted to dairy cattle.

The cgMLST analysis further supported the placement of the Kazakhstan isolate within the bovine-associated ST151 population. The closest cgMLST profile was cgST32654, with 1,654 of 1,716 cgMLST loci matched and 62 allelic differences. This result indicates that the isolate is closely related to existing ST151 profiles in the PubMLST database, while still showing allelic divergence consistent with a distinct regional isolate. The circular cgMLST tree showed that ST151 isolates were largely represented by animal- and milk-associated isolates from Europe, North America, and other regions, including Ireland, the United Kingdom, the Netherlands, Canada, the USA, Denmark, New Zealand, and Switzerland. This clustering pattern is consistent with previous reports describing ST151 as a bovine-adapted lineage associated with mastitis and dairy production systems (11, 17, 22, 23).

The cgMLST allelic comparison of the Kazakhstan isolate has several implications. First, it suggests that the studied Kazakhstan isolate is allelically related to publicly available bovine-associated ST151 profiles rather than representing an isolated genomic background. Second, it highlights the value of cgMLST for placing regional isolates into an international genomic context. Third, it supports the need for broader genomic surveillance of *S. aureus* in Kazakhstan and Central Asia to determine whether ST151 is sporadic, locally established, or part of a wider regional dairy-associated population. Because the present study analyzed a single isolate, population-level conclusions cannot yet be made. However, the data provide an important baseline for future comparative studies involving larger numbers of isolates from different farms, regions, hosts, and time periods.

The virulence gene profile of the isolate is consistent with the presence of genomic loci commonly associated with mastitis-associated *Staphylococcus aureus* (2, 4, 24). The detected genes included loci encoding hemolysins, leukocidins, proteases, and egc-associated enterotoxin-like proteins. Genes such as hlgA, hlgB, hlgC, lukD, lukE, aur, splA, and splB may contribute to host cell damage, inflammatory responses, and host-pathogen interaction. The genes seg, sei, sem, sen, seo, and seu were interpreted as egc-associated enterotoxin-like genes. Their detection should not be considered evidence of toxin expression, toxin production, or food poisoning risk, because these phenotypes were not experimentally assessed in the present study.

The histopathological findings are consistent with the virulence-associated genomic profile, although they should be interpreted as complementary case-level evidence rather than direct functional validation of gene expression. Lesions consistent with acute suppurative mastitis, including inflammatory infiltration, tissue damage, and disruption of mammary architecture, are compatible with the pathogenic mechanisms mediated by cytolysins, leukotoxins, proteases, and other virulence factors. Previous studies have shown that *S. aureus* can cause extensive mammary tissue damage through toxin-mediated cytotoxicity, immune cell recruitment, and persistence within host tissues (25, 26). The combination of histopathology and WGS-based virulence profiling therefore provides complementary evidence for interpreting the pathogenic potential of this single isolate.

Antimicrobial resistance is another important aspect of mastitis-associated *S. aureus*. In Kazakhstan and other regions, resistance to commonly used antimicrobials has been reported among *S. aureus* isolates from bovine milk and mastitis cases (14, 15). In the present study, CARD RGI detected several AMR-associated efflux and regulatory loci, including norA, norC, sdrM, sepA, LmrS, mgrA, mepR, kdpD, and arlR, as well as target-alteration-related markers. However, these findings were interpreted cautiously because many of these loci are chromosomal, intrinsic, regulatory, or efflux-associated and do not by themselves establish categorical antimicrobial resistance or multidrug resistance. Therefore, phenotypic antimicrobial susceptibility testing remains necessary for categorical resistance interpretation (8, 27).

Importantly, ResFinder did not detect acquired antimicrobial resistance genes, and PointFinder did not identify known resistance-associated chromosomal point mutations. Therefore, the isolate was not classified as MRSA, and no mecA-, SCCmec-, or mec-complex-based interpretation was made. Genomic prediction alone does not always fully explain phenotypic antimicrobial resistance, and genotype–phenotype discordance has been reported for some resistance and virulence markers (28, 29). Therefore, WGS-based resistance prediction should be interpreted together with phenotypic antimicrobial susceptibility testing.

The integrated approach used in this study is particularly important for pathogens such as *S. aureus*, where pathogenicity and resistance are multifactorial. WGS provides a comprehensive overview of genetic determinants, but experimental and phenotypic data remain necessary to interpret biological relevance. This is consistent with previous studies emphasizing that WGS should complement, rather than fully replace, conventional microbiological testing, antimicrobial susceptibility testing, and histopathological assessment (30, 31). In this context, the combination of WGS, MLST, cgMLST, virulence gene detection, antimicrobial resistance profiling, and pathological assessment provides a more complete understanding of the isolate than any single method alone.

The study also confirms the continued relevance of conventional molecular tools such as species-specific *nuc* PCR. Although WGS provides superior resolution for genotyping and phylogenomic analysis, rapid PCR-based confirmation remains valuable for routine diagnostics, especially in resource-limited veterinary laboratories. The use of *nuc* gene detection for species confirmation remains widely reported in studies of bovine mastitis-associated *S. aureus* and continues to provide a cost-effective and specific diagnostic approach (32–35). Thus, combining rapid molecular confirmation with WGS-based characterization offers a practical framework for both routine diagnosis and advanced epidemiological investigation.

From a One Health perspective, the findings are relevant because *S. aureus* can circulate among animals, humans, food products, and the farm environment. Livestock-associated lineages, including methicillin-resistant and multidrug-resistant strains, have been increasingly recognized as potential threats to both animal and human health (36, 37). Although the present study focused on a bovine mastitis-associated isolate, the detection of virulence and resistance-associated genes highlights the importance of surveillance at the animal-human-environment interface. Comparative genomic studies involving isolates from cattle, milk, farm workers, and dairy environments would help clarify transmission routes, host adaptation, and possible zoonotic risks in Kazakhstan and the broader Central Asian region.

The regional relevance of this work is important. Previous studies from Kazakhstan have reported *S. aureus* contamination in raw cow milk and mastitis-associated samples, including phenotypic resistance to *β*-lactams and tetracyclines (14, 15). However, detailed WGS-based lineage assignment and comparative cgMLST analysis have remained limited. By characterizing a single ST151 isolate and comparing it with public cgMLST profiles, this study contributes to filling that gap. The findings support the implementation of genomic surveillance for mastitis-associated pathogens in Kazakhstan, particularly for monitoring antimicrobial resistance, virulence profiles, and the introduction or persistence of globally distributed bovine-associated lineages.

This work also aligns with recent studies from Kazakhstan demonstrating the increasing use of molecular, serological, and epidemiological approaches for livestock disease surveillance in the region (38–40). Together with these efforts, the present WGS-based characterization of bovine mastitis-associated *S. aureus* contributes to the broader development of evidence-based animal disease monitoring, antimicrobial stewardship, and control strategies in Kazakhstan and Central Asia.

This study has limitations. First, only one *S. aureus* isolate was analyzed, and therefore the findings cannot be used to estimate the prevalence of ST151 or other lineages in Kazakhstan. Second, while cgMLST comparison provides comparative allelic context, the available public metadata were incomplete for some isolates, which may limit detailed interpretation of geographic and epidemiological clustering. Third, detection of virulence and antimicrobial resistance genes does not necessarily confirm their expression or phenotypic impact. Fourth, although oxacillin disk diffusion screening was performed, the assay was not conducted under a fully standardized antimicrobial susceptibility testing system. Therefore, the oxacillin result was interpreted only as supportive screening evidence and not as formal confirmation of MRSA status. Fifth, SCCmec type, detailed mec-complex structure, mobile genetic element context, and plasmid localization of resistance-associated loci were not resolved. Finally, because the genome was generated using long-read sequencing and no independent polishing step could be confirmed, small-scale genomic details, especially those related to resistance genes and mobile genetic elements, should be interpreted cautiously. Future studies should include larger isolate collections, phenotypic antimicrobial susceptibility testing, biofilm assays, gene expression analysis, and longitudinal sampling from multiple farms and regions. Such studies would help determine whether ST151 is widely distributed in Kazakhstan and clarify its role in persistent mastitis, antimicrobial resistance dissemination, and possible zoonotic transmission.

Overall, this study demonstrates the value of integrating conventional diagnostics, histopathology, whole-genome sequencing, MLST, cgMLST, and antimicrobial resistance and virulence gene profiling for the characterization of bovine mastitis-associated *S. aureus*. The identification of a Kazakhstan isolate assigned to ST151 provides a useful reference point for future comparative genomic studies of mastitis-associated *S. aureus* in Central Asia. Although the study was limited to a single isolate, the findings establish an important genomic baseline for future surveillance and support the development of evidence-based mastitis control, antimicrobial stewardship, and One Health monitoring strategies in the region.

## 5 Conclusion

This study presents a single-isolate genomic characterization of a bovine mastitis-associated *Staphylococcus aureus* ST151 isolate from Kazakhstan. The isolate was recovered from a severe clinical mastitis case, confirmed as *S. aureus* by bacteriological and nuc gene PCR methods, and characterized using whole-genome sequencing, MLST, cgMLST, virulence gene profiling, antimicrobial resistance gene analysis, supportive phenotypic antimicrobial susceptibility screening, experimental virulence assessment, and histopathological examination.

The isolate belonged to ST151 and showed cgMLST allelic relatedness to publicly available bovine-associated ST151 profiles. The genome also carried virulence-associated genes, including exoenzyme, hemolysin, leukocidin, and egc-associated enterotoxin-like genes. CARD RGI detected several AMR-associated efflux and regulatory loci; however, ResFinder did not detect acquired antimicrobial resistance genes, and PointFinder did not identify known resistance-associated point mutations. Therefore, the antimicrobial resistance findings were interpreted cautiously and not as evidence of multidrug resistance or MRSA status.

Because this study analyzed only one selected isolate, the findings cannot be used to determine the prevalence, distribution, or epidemiological dominance of ST151 in Kazakhstan. Instead, the genome provides a useful reference point for future genomic surveillance and comparative studies of mastitis-associated *S. aureus* in Kazakhstan and Central Asia. Larger isolate collections from multiple farms, regions, hosts, and time periods will be required to clarify the local diversity, antimicrobial resistance patterns, and epidemiological significance of ST151 and other *S. aureus* lineages in the region.

## Data Availability

The genome sequence data generated in this study have been deposited in the DDBJ/ENA/GenBank database under WGS accession number JBYFBM000000000. The version described in this paper is JBYFBM010000000. The sequencing project is available under BioProject accession number PRJNA1465568 and the corresponding BioSample accession number is SAMN59747580.
