# Supplementary material for: Single-isolate genomic characterization and cgMLST allelic comparison of a bovine mastitis-associated Staphylococcus aureus ST151 isolate from Kazakhstan
Source: Front Vet Sci. 2026 Jul 30;13:1881180. doi: 10.3389/fvets.2026.1881180 (PMC13467824; doi:10.3389/fvets.2026.1881180)
Supplement: Supplementary file 1 [file Table_1.docx]

**Supplementary Table S1.**

**Initial PubMLST ST151 metadata export and filtering summary for the cgMLST minimum-spanning tree analysis.**

The table includes public ST151 records retrieved from PubMLST, cgMLST data availability, metadata availability, and whether each record was retained for the final Figure 5 analysis. Records without sufficient cgMLST allelic profile data were excluded from the final minimum-spanning tree.

| **No.** | **PubMLST isolate ID** | **Isolate name** | **ST (MLST)** | **cgST (cgMLST)** | **Country** | **Year** | **Disease** | **Source** | **cgMLST loci called (of 1,716)** | **Included in high-coverage cgMLST candidate set (≥1,654 loci)** | **Allelic distance to SA_KSRVI_2025_1** | **Metadata availability** | **Notes** |
| --- | --- | --- | --- | --- | --- | --- | --- | --- | --- | --- | --- | --- | --- |
| 1 | 1081 | ESB1 | 151 | not available in export | UK | 1998 | invasive (unspecified/other) | unknown | 0 | no | not available in export | partial | Public PubMLST ST151 record |
| 2 | 1786 | PP433 | 151 | not available in export | UK [England] | 2006 | none (food/environmental) | milk | 0 | no | not available in export | complete | Public PubMLST ST151 record |
| 3 | 2714 | 19 | 151 | not available in export | The Netherlands | 2007 | mastitis | milk | 0 | no | not available in export | complete | Public PubMLST ST151 record |
| 4 | 2727 | 34 | 151 | not available in export | The Netherlands | 2007 | mastitis | milk | 0 | no | not available in export | complete | Public PubMLST ST151 record |
| 5 | 2733 | 40 | 151 | not available in export | The Netherlands | 2007 | mastitis | milk | 0 | no | not available in export | complete | Public PubMLST ST151 record |
| 6 | 2817 | 322 | 151 | not available in export | The Netherlands | 2007 | mastitis | milk | 0 | no | not available in export | complete | Public PubMLST ST151 record |
| 7 | 2854 | 369 | 151 | not available in export | The Netherlands | 2007 | mastitis | milk | 0 | no | not available in export | complete | Public PubMLST ST151 record |
| 8 | 2855 | 370 | 151 | not available in export | The Netherlands | 2007 | mastitis | milk | 0 | no | not available in export | complete | Public PubMLST ST151 record |
| 9 | 2856 | 371 | 151 | not available in export | The Netherlands | 2007 | mastitis | milk | 0 | no | not available in export | complete | Public PubMLST ST151 record |
| 10 | 2903 | 470 | 151 | not available in export | The Netherlands | 2007 | mastitis | milk | 0 | no | not available in export | complete | Public PubMLST ST151 record |
| 11 | 2904 | 472 | 151 | not available in export | The Netherlands | 2008 | mastitis | milk | 0 | no | not available in export | complete | Public PubMLST ST151 record |
| 12 | 3111 | MOK034 | 151 | not available in export | Ireland | 2010 | mastitis | animal | 0 | no | not available in export | complete | Public PubMLST ST151 record |
| 13 | 3113 | MOK037 | 151 | not available in export | Ireland | 2010 | mastitis | animal | 0 | no | not available in export | complete | Public PubMLST ST151 record |
| 14 | 3118 | MOK043 | 151 | not available in export | Ireland | 2010 | mastitis | animal | 0 | no | not available in export | complete | Public PubMLST ST151 record |
| 15 | 3122 | MOK048 | 151 | not available in export | Ireland | 2010 | mastitis | animal | 0 | no | not available in export | complete | Public PubMLST ST151 record |
| 16 | 3124 | MOK050 | 151 | not available in export | Ireland | 2011 | mastitis | animal | 0 | no | not available in export | complete | Public PubMLST ST151 record |
| 17 | 3130 | MOK056 | 151 | not available in export | Ireland | 2010 | mastitis | animal | 0 | no | not available in export | complete | Public PubMLST ST151 record |
| 18 | 3131 | MOK058 | 151 | not available in export | Ireland | 2010 | mastitis | animal | 0 | no | not available in export | complete | Public PubMLST ST151 record |
| 19 | 3137 | MOK065 | 151 | not available in export | Ireland | 2010 | mastitis | animal | 0 | no | not available in export | complete | Public PubMLST ST151 record |
| 20 | 3138 | MOK066 | 151 | not available in export | Ireland | 2010 | mastitis | animal | 0 | no | not available in export | complete | Public PubMLST ST151 record |
| 21 | 3139 | MOK067 | 151 | not available in export | Ireland | 2010 | mastitis | animal | 0 | no | not available in export | complete | Public PubMLST ST151 record |
| 22 | 3142 | MOK070 | 151 | not available in export | Ireland | 2010 | mastitis | animal | 0 | no | not available in export | complete | Public PubMLST ST151 record |
| 23 | 3143 | MOK071 | 151 | not available in export | Ireland | 2010 | mastitis | animal | 0 | no | not available in export | complete | Public PubMLST ST151 record |
| 24 | 3161 | MOK092 | 151 | not available in export | Ireland | 2010 | mastitis | animal | 0 | no | not available in export | complete | Public PubMLST ST151 record |
| 25 | 3162 | MOK093 | 151 | not available in export | Ireland | 2010 | mastitis | animal | 0 | no | not available in export | complete | Public PubMLST ST151 record |
| 26 | 3163 | MOK094 | 151 | not available in export | Ireland | 2010 | mastitis | animal | 0 | no | not available in export | complete | Public PubMLST ST151 record |
| 27 | 3164 | MOK096 | 151 | not available in export | Ireland | 2010 | mastitis | animal | 0 | no | not available in export | complete | Public PubMLST ST151 record |
| 28 | 3171 | MOK103 | 151 | not available in export | Ireland | 2010 | mastitis | animal | 0 | no | not available in export | complete | Public PubMLST ST151 record |
| 29 | 3182 | MOK118 | 151 | not available in export | Ireland | 2011 | mastitis | animal | 0 | no | not available in export | complete | Public PubMLST ST151 record |
| 30 | 3184 | MOK120 | 151 | not available in export | Ireland | 2011 | mastitis | animal | 0 | no | not available in export | complete | Public PubMLST ST151 record |
| 31 | 3185 | MOK121 | 151 | not available in export | Ireland | 2010 | mastitis | animal | 0 | no | not available in export | complete | Public PubMLST ST151 record |
| 32 | 3193 | SA1 | 151 | not available in export | Spain | 2007 | mastitis | milk | 0 | no | not available in export | complete | Public PubMLST ST151 record |
| 33 | 3195 | SA4 | 151 | not available in export | Spain | 2007 | mastitis | milk | 0 | no | not available in export | complete | Public PubMLST ST151 record |
| 34 | 3217 | GDC6 | 151 | not available in export | Spain | 2007 | carrier | milk | 0 | no | not available in export | complete | Public PubMLST ST151 record |
| 35 | 3228 | MOK124 | 151 | not available in export | Ireland | 2011 | mastitis | animal | 0 | no | not available in export | complete | Public PubMLST ST151 record |
| 36 | 3231 | MOK127 | 151 | not available in export | Ireland | 2011 | mastitis | animal | 0 | no | not available in export | complete | Public PubMLST ST151 record |
| 37 | 3234 | MOK131 | 151 | not available in export | Ireland | 2010 | mastitis | animal | 0 | no | not available in export | complete | Public PubMLST ST151 record |
| 38 | 3237 | MOK134 | 151 | not available in export | Ireland | 2010 | mastitis | animal | 0 | no | not available in export | complete | Public PubMLST ST151 record |
| 39 | 3253 | MOK137 | 151 | not available in export | Ireland | 2010 | mastitis | animal | 0 | no | not available in export | complete | Public PubMLST ST151 record |
| 40 | 3254 | MOK138 | 151 | not available in export | Ireland | 2010 | mastitis | animal | 0 | no | not available in export | complete | Public PubMLST ST151 record |
| 41 | 3526 | MOK205 | 151 | not available in export | Ireland | 2010 | mastitis | animal | 0 | no | not available in export | complete | Public PubMLST ST151 record |
| 42 | 3527 | MOK206 | 151 | not available in export | Ireland | 2010 | mastitis | animal | 0 | no | not available in export | complete | Public PubMLST ST151 record |
| 43 | 4329 | SP2208-2 | 151 | not available in export | USA | 2004 | mastitis | milk | 0 | no | not available in export | complete | Public PubMLST ST151 record |
| 44 | 4330 | SP309-1 | 151 | not available in export | USA | 2004 | mastitis | milk | 0 | no | not available in export | complete | Public PubMLST ST151 record |
| 45 | 4347 | CR3060-3 | 151 | not available in export | USA | 2006 | mastitis | milk | 0 | no | not available in export | complete | Public PubMLST ST151 record |
| 46 | 4348 | CR3200-1 | 151 | not available in export | USA | 2006 | mastitis | milk | 0 | no | not available in export | complete | Public PubMLST ST151 record |
| 47 | 5716 | OF2-02 | 151 | not available in export | USA | 2013 | unknown | milk | 0 | no | not available in export | partial | Public PubMLST ST151 record |
| 48 | 5717 | OF43-02 | 151 | not available in export | USA | 2013 | unknown | milk | 0 | no | not available in export | partial | Public PubMLST ST151 record |
| 49 | 5718 | OF34-02 | 151 | not available in export | USA | 2013 | unknown | milk | 0 | no | not available in export | partial | Public PubMLST ST151 record |
| 50 | 5719 | OF11-05 | 151 | not available in export | USA | 2013 | unknown | milk | 0 | no | not available in export | partial | Public PubMLST ST151 record |
| 51 | 5720 | OF35-02 | 151 | not available in export | USA | 2013 | unknown | milk | 0 | no | not available in export | partial | Public PubMLST ST151 record |
| 52 | 5721 | OF7-02 | 151 | not available in export | USA | 2013 | unknown | milk | 0 | no | not available in export | partial | Public PubMLST ST151 record |
| 53 | 5722 | OF31-05 | 151 | not available in export | USA | 2013 | unknown | milk | 0 | no | not available in export | partial | Public PubMLST ST151 record |
| 54 | 5723 | OF36-09 | 151 | not available in export | USA | 2013 | unknown | milk | 0 | no | not available in export | partial | Public PubMLST ST151 record |
| 55 | 5724 | OF36-01 | 151 | not available in export | USA | 2013 | unknown | milk | 0 | no | not available in export | partial | Public PubMLST ST151 record |
| 56 | 5725 | OF33-04 | 151 | not available in export | USA | 2013 | unknown | milk | 0 | no | not available in export | partial | Public PubMLST ST151 record |
| 57 | 5726 | OF29-01 | 151 | not available in export | USA | 2013 | unknown | milk | 0 | no | not available in export | partial | Public PubMLST ST151 record |
| 58 | 5727 | OF42-05 | 151 | not available in export | USA | 2013 | unknown | milk | 0 | no | not available in export | partial | Public PubMLST ST151 record |
| 59 | 5728 | OF44-05 | 151 | not available in export | USA | 2013 | unknown | milk | 0 | no | not available in export | partial | Public PubMLST ST151 record |
| 60 | 5729 | OF26-11 | 151 | not available in export | USA | 2013 | unknown | milk | 0 | no | not available in export | partial | Public PubMLST ST151 record |
| 61 | 5730 | OF10-09 | 151 | not available in export | USA | 2013 | unknown | milk | 0 | no | not available in export | partial | Public PubMLST ST151 record |
| 62 | 5731 | OF44-07 | 151 | not available in export | USA | 2013 | unknown | milk | 0 | no | not available in export | partial | Public PubMLST ST151 record |
| 63 | 5732 | OF17-30 | 151 | not available in export | USA | 2013 | unknown | milk | 0 | no | not available in export | partial | Public PubMLST ST151 record |
| 64 | 5969 | MOK008 | 151 | not available in export | Ireland | 2010 | mastitis | animal | 0 | no | not available in export | complete | Public PubMLST ST151 record |
| 65 | 5972 | MOK013 | 151 | not available in export | Ireland | 2010 | mastitis | animal | 0 | no | not available in export | complete | Public PubMLST ST151 record |
| 66 | 5974 | MOK017 | 151 | not available in export | Ireland | 2010 | mastitis | animal | 0 | no | not available in export | complete | Public PubMLST ST151 record |
| 67 | 8934 | ERR084616 | 151 | not available in export | Unknown |  | unknown | unknown | 1695 | yes | not available in export | not available | Public PubMLST ST151 record |
| 68 | 8935 | ERR084624 | 151 | not available in export | Unknown |  | unknown | unknown | 1694 | yes | not available in export | not available | Public PubMLST ST151 record |
| 69 | 8975 | ERR084625 | 151 | not available in export | Unknown |  | unknown | unknown | 1695 | yes | not available in export | not available | Public PubMLST ST151 record |
| 70 | 9004 | ERR084620 | 151 | not available in export | Unknown |  | unknown | unknown | 1696 | yes | not available in export | not available | Public PubMLST ST151 record |
| 71 | 14444 | ERR246595 | 151 | not available in export | UK | 2012 | unknown | unknown | 1693 | yes | not available in export | partial | Public PubMLST ST151 record |
| 72 | 14454 | ERR246611 | 151 | not available in export | UK | 2012 | unknown | unknown | 1697 | yes | not available in export | partial | Public PubMLST ST151 record |
| 73 | 14457 | ERR246615 | 151 | not available in export | UK | 2012 | unknown | unknown | 1697 | yes | not available in export | partial | Public PubMLST ST151 record |
| 74 | 14464 | ERR246624 | 151 | not available in export | UK | 2012 | unknown | unknown | 1693 | yes | not available in export | partial | Public PubMLST ST151 record |
| 75 | 14472 | ERR246634 | 151 | not available in export | Unknown |  | unknown | unknown | 1688 | yes | not available in export | not available | Public PubMLST ST151 record |
| 76 | 14477 | ERR246639 | 151 | not available in export | UK | 2012 | unknown | unknown | 1696 | yes | not available in export | partial | Public PubMLST ST151 record |
| 77 | 14478 | ERR246640 | 151 | not available in export | UK | 2012 | unknown | unknown | 1693 | yes | not available in export | partial | Public PubMLST ST151 record |
| 78 | 14479 | ERR246641 | 151 | not available in export | UK | 2012 | unknown | unknown | 1694 | yes | not available in export | partial | Public PubMLST ST151 record |
| 79 | 14484 | ERR246647 | 151 | not available in export | UK | 2012 | unknown | unknown | 1694 | yes | not available in export | partial | Public PubMLST ST151 record |
| 80 | 14497 | ERR246663 | 151 | not available in export | UK | 2012 | unknown | unknown | 1693 | yes | not available in export | partial | Public PubMLST ST151 record |
| 81 | 14499 | ERR246665 | 151 | not available in export | UK | 2012 | unknown | unknown | 1696 | yes | not available in export | partial | Public PubMLST ST151 record |
| 82 | 14503 | ERR246670 | 151 | not available in export | UK | 2012 | unknown | unknown | 1694 | yes | not available in export | partial | Public PubMLST ST151 record |
| 83 | 14504 | ERR246671 | 151 | not available in export | UK | 2012 | unknown | unknown | 1694 | yes | not available in export | partial | Public PubMLST ST151 record |
| 84 | 15068 | ERR246629 | 151 | not available in export | UK | 2012 | unknown | unknown | 1694 | yes | not available in export | partial | Public PubMLST ST151 record |
| 85 | 15375 | ERR246597 | 151 | not available in export | UK | 2012 | unknown | unknown | 1693 | yes | not available in export | partial | Public PubMLST ST151 record |
| 86 | 15377 | ERR246602 | 151 | not available in export | UK | 2012 | unknown | unknown | 1696 | yes | not available in export | partial | Public PubMLST ST151 record |
| 87 | 21607 | ERR387198 | 151 | not available in export | Unknown |  | unknown | unknown | 1696 | yes | not available in export | not available | Public PubMLST ST151 record |
| 88 | 21608 | ERR387200 | 151 | not available in export | Unknown |  | unknown | unknown | 1696 | yes | not available in export | not available | Public PubMLST ST151 record |
| 89 | 21609 | ERR387202 | 151 | not available in export | Unknown |  | unknown | unknown | 1696 | yes | not available in export | not available | Public PubMLST ST151 record |
| 90 | 22212 | ERR387201 | 151 | not available in export | Unknown |  | unknown | unknown | 1696 | yes | not available in export | not available | Public PubMLST ST151 record |
| 91 | 29893 | ERR387199 | 151 | not available in export | Unknown |  | unknown | unknown | 1696 | yes | not available in export | not available | Public PubMLST ST151 record |
| 92 | 31152 | ERR845409 | 151 | not available in export | Unknown |  | unknown | unknown | 1693 | yes | not available in export | not available | Public PubMLST ST151 record |
| 93 | 31196 | ERR845504 | 151 | not available in export | Unknown |  | unknown | unknown | 1694 | yes | not available in export | not available | Public PubMLST ST151 record |
| 94 | 34934 | AC06-23A | 151 | not available in export | USA | 2018 | none (food/environmental) | milk | 0 | no | not available in export | complete | Public PubMLST ST151 record |
| 95 | 34935 | AC06-23B | 151 | not available in export | USA | 2018 | none (food/environmental) | milk | 0 | no | not available in export | complete | Public PubMLST ST151 record |
| 96 | 34936 | AC06-23C | 151 | not available in export | USA | 2018 | none (food/environmental) | milk | 0 | no | not available in export | complete | Public PubMLST ST151 record |
| 97 | 34942 | AC07-36 | 151 | not available in export | USA | 2018 | mastitis | milk | 0 | no | not available in export | complete | Public PubMLST ST151 record |
| 98 | 34945 | AC07-53 | 151 | not available in export | USA | 2018 | none (food/environmental) | milk | 0 | no | not available in export | complete | Public PubMLST ST151 record |
| 99 | 34946 | AC07-57 | 151 | not available in export | USA | 2018 | none (food/environmental) | milk | 0 | no | not available in export | complete | Public PubMLST ST151 record |
| 100 | 34947 | AC07-59 | 151 | not available in export | USA | 2018 | none (food/environmental) | milk | 0 | no | not available in export | complete | Public PubMLST ST151 record |
| 101 | 34948 | AC07-60 | 151 | not available in export | USA | 2018 | none (food/environmental) | milk | 0 | no | not available in export | complete | Public PubMLST ST151 record |
| 102 | 34949 | AC07-67 | 151 | not available in export | USA | 2018 | mastitis | milk | 0 | no | not available in export | complete | Public PubMLST ST151 record |
| 103 | 34950 | AC07-68 | 151 | not available in export | USA | 2018 | mastitis | milk | 0 | no | not available in export | complete | Public PubMLST ST151 record |
| 104 | 34951 | AC07-71 | 151 | not available in export | USA | 2018 | mastitis | milk | 0 | no | not available in export | complete | Public PubMLST ST151 record |
| 105 | 34952 | AC07-73 | 151 | not available in export | USA | 2018 | mastitis | milk | 0 | no | not available in export | complete | Public PubMLST ST151 record |
| 106 | 34953 | AC07-75 | 151 | not available in export | USA | 2018 | none (food/environmental) | milk | 0 | no | not available in export | complete | Public PubMLST ST151 record |
| 107 | 34954 | AC07-78 | 151 | not available in export | USA | 2018 | none (food/environmental) | milk | 0 | no | not available in export | complete | Public PubMLST ST151 record |
| 108 | 34997 | AC14-44 | 151 | not available in export | USA | 2018 | none (food/environmental) | milk | 0 | no | not available in export | complete | Public PubMLST ST151 record |
| 109 | 34998 | AC14-45 | 151 | not available in export | USA | 2018 | none (food/environmental) | milk | 0 | no | not available in export | complete | Public PubMLST ST151 record |
| 110 | 35000 | AC15-08 | 151 | not available in export | USA | 2018 | none (food/environmental) | milk | 0 | no | not available in export | complete | Public PubMLST ST151 record |
| 111 | 35002 | AC15-20 | 151 | not available in export | USA | 2018 | none (food/environmental) | milk | 0 | no | not available in export | complete | Public PubMLST ST151 record |
| 112 | 35004 | AC15-66 | 151 | not available in export | USA | 2018 | mastitis | milk | 0 | no | not available in export | complete | Public PubMLST ST151 record |
| 113 | 35005 | AC15-67 | 151 | not available in export | USA | 2018 | mastitis | milk | 0 | no | not available in export | complete | Public PubMLST ST151 record |
| 114 | 35014 | AC17-47 | 151 | not available in export | USA | 2018 | carrier | skin swab | 0 | no | not available in export | complete | Public PubMLST ST151 record |
| 115 | 35018 | AC28-6 | 151 | not available in export | USA | 2018 | mastitis | milk | 0 | no | not available in export | complete | Public PubMLST ST151 record |
| 116 | 35019 | AC28-15 | 151 | not available in export | USA | 2018 | mastitis | milk | 0 | no | not available in export | complete | Public PubMLST ST151 record |
| 117 | 35020 | AC28-20 | 151 | not available in export | USA | 2018 | mastitis | milk | 0 | no | not available in export | complete | Public PubMLST ST151 record |
| 118 | 35021 | AC28-21 | 151 | not available in export | USA | 2018 | mastitis | milk | 0 | no | not available in export | complete | Public PubMLST ST151 record |
| 119 | 35022 | AC28-46 | 151 | not available in export | USA | 2018 | none (food/environmental) | milk | 0 | no | not available in export | complete | Public PubMLST ST151 record |
| 120 | 39194 | RF122 | 151 | not available in export | Ireland | 2002 | mastitis | milk | 1680 | yes | not available in export | complete | Public PubMLST ST151 record |
| 121 | 42199 | 114 | 151 | not available in export | UK |  | other | animal | 1693 | yes | not available in export | partial | Public PubMLST ST151 record |
| 122 | 42203 | 38963 | 151 | not available in export | UK |  | other | animal | 1697 | yes | not available in export | partial | Public PubMLST ST151 record |
| 123 | 42217 | B119 | 151 | not available in export | Ireland | 1998 | other | animal | 1693 | yes | not available in export | complete | Public PubMLST ST151 record |
| 124 | 42219 | B130 | 151 | not available in export | Ireland | 1998 | other | animal | 1696 | yes | not available in export | complete | Public PubMLST ST151 record |
| 125 | 42227 | CI2315 | 151 | not available in export | UK |  | other | animal | 1696 | yes | not available in export | partial | Public PubMLST ST151 record |
| 126 | 42333 | C05_232 | 151 | not available in export | UK [England] | 2006 | unknown | animal | 1695 | yes | not available in export | partial | Public PubMLST ST151 record |
| 127 | 42339 | C04_847 | 151 | not available in export | UK [England] | 2006 | unknown | animal | 1695 | yes | not available in export | partial | Public PubMLST ST151 record |
| 128 | 42528 | NCTC9553 | 151 | not available in export | UK | 1954 | unknown | animal | 1698 | yes | not available in export | partial | Public PubMLST ST151 record |
| 129 | 42720 | 909 | 151 | not available in export | The Netherlands | 2014 | unknown | milk | 1694 | yes | not available in export | partial | Public PubMLST ST151 record |
| 130 | 42805 | SAU_0023 | 151 | not available in export | Canada | 2007 | unknown | milk | 1676 | yes | not available in export | partial | Public PubMLST ST151 record |
| 131 | 42807 | SAU_0462 | 151 | not available in export | Canada | 2007 | unknown | milk | 1648 | no | not available in export | partial | Public PubMLST ST151 record |
| 132 | 42810 | SAU_2961 | 151 | not available in export | Canada | 2007 | unknown | milk | 1632 | no | not available in export | partial | Public PubMLST ST151 record |
| 133 | 42811 | SAU_1021 | 151 | not available in export | Canada | 2007 | unknown | milk | 1645 | no | not available in export | partial | Public PubMLST ST151 record |
| 134 | 42816 | SAU_1997 | 151 | not available in export | Canada | 2007 | unknown | milk | 1598 | no | not available in export | partial | Public PubMLST ST151 record |
| 135 | 42819 | SAU_2872 | 151 | not available in export | Canada | 2007 | unknown | milk | 1639 | no | not available in export | partial | Public PubMLST ST151 record |
| 136 | 42823 | SAU_1482 | 151 | not available in export | Canada | 2007 | unknown | milk | 1601 | no | not available in export | partial | Public PubMLST ST151 record |
| 137 | 42828 | SAU_4415 | 151 | not available in export | Canada | 2007 | unknown | milk | 1569 | no | not available in export | partial | Public PubMLST ST151 record |
| 138 | 42832 | SAU_4554 | 151 | not available in export | Canada | 2007 | unknown | milk | 1606 | no | not available in export | partial | Public PubMLST ST151 record |
| 139 | 42833 | SAU_6049 | 151 | not available in export | Canada | 2007 | unknown | milk | 1585 | no | not available in export | partial | Public PubMLST ST151 record |
| 140 | 42834 | SAU_0027 | 151 | not available in export | Canada | 2007 | unknown | milk | 1643 | no | not available in export | partial | Public PubMLST ST151 record |
| 141 | 42840 | SAU_0362 | 151 | not available in export | Canada | 2007 | unknown | milk | 1679 | yes | not available in export | partial | Public PubMLST ST151 record |
| 142 | 42844 | SAU_1132a | 151 | not available in export | Canada | 2007 | unknown | milk | 1625 | no | not available in export | partial | Public PubMLST ST151 record |
| 143 | 42846 | SAU_1212 | 151 | not available in export | Canada | 2008 | unknown | milk | 1637 | no | not available in export | partial | Public PubMLST ST151 record |
| 144 | 42847 | SAU_1779 | 151 | not available in export | Canada | 2008 | unknown | milk | 1613 | no | not available in export | partial | Public PubMLST ST151 record |
| 145 | 42848 | SAU_1894 | 151 | not available in export | Canada | 2007 | unknown | milk | 1657 | yes | not available in export | partial | Public PubMLST ST151 record |
| 146 | 42850 | SAU_2027 | 151 | not available in export | Canada | 2007 | unknown | milk | 1635 | no | not available in export | partial | Public PubMLST ST151 record |
| 147 | 42852 | SAU_2526 | 151 | not available in export | Canada | 2008 | unknown | milk | 1601 | no | not available in export | partial | Public PubMLST ST151 record |
| 148 | 42854 | SAU_2984 | 151 | not available in export | Canada | 2007 | unknown | milk | 1669 | yes | not available in export | partial | Public PubMLST ST151 record |
| 149 | 42856 | SAU_3457 | 151 | not available in export | Canada | 2007 | unknown | milk | 1675 | yes | not available in export | partial | Public PubMLST ST151 record |
| 150 | 42857 | SAU_3559 | 151 | not available in export | Canada | 2007 | unknown | milk | 1589 | no | not available in export | partial | Public PubMLST ST151 record |
| 151 | 42859 | SAU_4178 | 151 | not available in export | Canada | 2007 | unknown | milk | 1669 | yes | not available in export | partial | Public PubMLST ST151 record |
| 152 | 42864 | SAU_5-SA | 151 | not available in export | Canada | 2007 | unknown | milk | 1654 | yes | not available in export | partial | Public PubMLST ST151 record |
| 153 | 42865 | SAU_7231 | 151 | not available in export | Canada | 2007 | unknown | milk | 1665 | yes | not available in export | partial | Public PubMLST ST151 record |
| 154 | 42866 | SAU_7526 | 151 | not available in export | Canada | 2007 | unknown | milk | 1601 | no | not available in export | partial | Public PubMLST ST151 record |
| 155 | 42871 | SAU_9309 | 151 | not available in export | Canada | 2007 | unknown | milk | 1631 | no | not available in export | partial | Public PubMLST ST151 record |
| 156 | 42876 | SAU_0799 | 151 | not available in export | Canada | 2007 | unknown | milk | 1680 | yes | not available in export | partial | Public PubMLST ST151 record |
| 157 | 42878 | SAU_1052 | 151 | not available in export | Canada | 2008 | unknown | milk | 1674 | yes | not available in export | partial | Public PubMLST ST151 record |
| 158 | 42884 | SAU_2025 | 151 | not available in export | Canada | 2007 | unknown | milk | 1679 | yes | not available in export | partial | Public PubMLST ST151 record |
| 159 | 42885 | SAU_2104 | 151 | not available in export | Canada | 2007 | unknown | milk | 1658 | yes | not available in export | partial | Public PubMLST ST151 record |
| 160 | 42887 | SAU_2811 | 151 | not available in export | Canada | 2008 | unknown | milk | 1670 | yes | not available in export | partial | Public PubMLST ST151 record |
| 161 | 42890 | SAU_9255 | 151 | not available in export | Canada | 2007 | unknown | milk | 1688 | yes | not available in export | partial | Public PubMLST ST151 record |
| 162 | 42895 | SAU_3057 | 151 | not available in export | Canada | 2008 | unknown | milk | 1663 | yes | not available in export | partial | Public PubMLST ST151 record |
| 163 | 42902 | SAU_0540 | 151 | not available in export | Canada | 2007 | unknown | milk | 1654 | yes | not available in export | partial | Public PubMLST ST151 record |
| 164 | 42907 | SAU_7404 | 151 | not available in export | Canada | 2007 | unknown | milk | 1579 | no | not available in export | partial | Public PubMLST ST151 record |
| 165 | 42910 | SAU_1205 | 151 | not available in export | Canada | 2007 | unknown | milk | 1680 | yes | not available in export | partial | Public PubMLST ST151 record |
| 166 | 42911 | SAU_1300 | 151 | not available in export | Canada | 2007 | unknown | milk | 1681 | yes | not available in export | partial | Public PubMLST ST151 record |
| 167 | 42914 | SAU_3205 | 151 | not available in export | Canada | 2007 | unknown | milk | 1672 | yes | not available in export | partial | Public PubMLST ST151 record |
| 168 | 42916 | SAU_6-SA | 151 | not available in export | Canada | 2007 | unknown | milk | 1683 | yes | not available in export | partial | Public PubMLST ST151 record |
| 169 | 42918 | SAU_8111 | 151 | not available in export | Canada | 2007 | unknown | milk | 1675 | yes | not available in export | partial | Public PubMLST ST151 record |
| 170 | 42920 | SAU_4506 | 151 | not available in export | Canada | 2007 | unknown | milk | 1687 | yes | not available in export | partial | Public PubMLST ST151 record |
| 171 | 42921 | SAU_7829 | 151 | not available in export | Canada | 2007 | unknown | milk | 1667 | yes | not available in export | partial | Public PubMLST ST151 record |
| 172 | 53389 | Sa253 | 151 | not available in export | Denmark | 2016 | unknown | animal | 1698 | yes | not available in export | partial | Public PubMLST ST151 record |
| 173 | 53393 | Sa113 | 151 | not available in export | Denmark | 2016 | unknown | animal | 1699 | yes | not available in export | partial | Public PubMLST ST151 record |
| 174 | 53396 | Sa98 | 151 | not available in export | Denmark | 2016 | unknown | animal | 1697 | yes | not available in export | partial | Public PubMLST ST151 record |
| 175 | 53401 | Sa92 | 151 | not available in export | Denmark | 2016 | unknown | animal | 1688 | yes | not available in export | partial | Public PubMLST ST151 record |
| 176 | 53402 | Sa99 | 151 | not available in export | Denmark | 2016 | unknown | animal | 1699 | yes | not available in export | partial | Public PubMLST ST151 record |
| 177 | 53410 | Sa101 | 151 | not available in export | Denmark | 2016 | unknown | animal | 1699 | yes | not available in export | partial | Public PubMLST ST151 record |
| 178 | 53417 | Sa88 | 151 | not available in export | Denmark | 2016 | unknown | animal | 1697 | yes | not available in export | partial | Public PubMLST ST151 record |
| 179 | 53418 | Sa71 | 151 | not available in export | Denmark | 2016 | unknown | animal | 1699 | yes | not available in export | partial | Public PubMLST ST151 record |
| 180 | 53421 | Sa58 | 151 | not available in export | Denmark | 2016 | unknown | animal | 1695 | yes | not available in export | partial | Public PubMLST ST151 record |
| 181 | 53431 | Sa65 | 151 | not available in export | Denmark | 2016 | unknown | animal | 1696 | yes | not available in export | partial | Public PubMLST ST151 record |
| 182 | 53456 | B130 | 151 | not available in export | Ireland | 1998 | unknown | animal | 1701 | yes | not available in export | partial | Public PubMLST ST151 record |
| 183 | 53487 | B119 | 151 | not available in export | Ireland | 1998 | unknown | animal | 1698 | yes | not available in export | partial | Public PubMLST ST151 record |
| 184 | 53688 | H03 | 151 | not available in export | Unknown | 2018 | unknown | animal | 1701 | yes | not available in export | partial | Public PubMLST ST151 record |
| 185 | 53691 | H06 | 151 | not available in export | Unknown | 2018 | unknown | animal | 1700 | yes | not available in export | partial | Public PubMLST ST151 record |
| 186 | 54400 | Sample_7415_2A_19 | 151 | not available in export | USA | 2019 | unknown | animal | 1696 | yes | not available in export | partial | Public PubMLST ST151 record |
| 187 | 54599 | ST151(DAPKQX01) | 151 | not available in export | USA | 2018 | unknown | animal | 1697 | yes | not available in export | partial | Public PubMLST ST151 record |
| 188 | 54600 | ST151(DAPKQY01) | 151 | not available in export | USA | 2018 | unknown | animal | 1698 | yes | not available in export | partial | Public PubMLST ST151 record |
| 189 | 54664 | Sample_6713_4a_18 | 151 | not available in export | USA | 2018 | unknown | animal | 1700 | yes | not available in export | partial | Public PubMLST ST151 record |
| 190 | 54675 | Sample_9361_1a_18 | 151 | not available in export | USA | 2019 | unknown | animal | 1699 | yes | not available in export | partial | Public PubMLST ST151 record |
| 191 | 54688 | Sample_2045A_18 | 151 | not available in export | USA | 2020 | unknown | animal | 1698 | yes | not available in export | partial | Public PubMLST ST151 record |
| 192 | 54721 | Sample_7856_33A_18 | 151 | not available in export | USA | 2018 | unknown | animal | 1700 | yes | not available in export | partial | Public PubMLST ST151 record |
| 193 | 54779 | Sample_8947A_17 | 151 | not available in export | USA | 2017 | unknown | animal | 1696 | yes | not available in export | partial | Public PubMLST ST151 record |
| 194 | 54945 | B123-056 | 151 | not available in export | New Zealand | 2019 | unknown | animal | 1703 | yes | not available in export | partial | Public PubMLST ST151 record |
| 195 | 54974 | B123-202-M | 151 | not available in export | New Zealand | 2019 | unknown | animal | 1702 | yes | not available in export | partial | Public PubMLST ST151 record |
| 196 | 54975 | B133-639 | 151 | not available in export | New Zealand | 2019 | unknown | animal | 1701 | yes | not available in export | partial | Public PubMLST ST151 record |
| 197 | 54976 | B113-551-M | 151 | not available in export | New Zealand | 2019 | unknown | animal | 1700 | yes | not available in export | partial | Public PubMLST ST151 record |
| 198 | 54992 | B123-045 | 151 | not available in export | New Zealand | 2019 | unknown | animal | 1703 | yes | not available in export | partial | Public PubMLST ST151 record |
| 199 | 55021 | 2009-10-84 | 151 | not available in export | Denmark | 2009 | unknown | animal | 1698 | yes | not available in export | partial | Public PubMLST ST151 record |
| 200 | 55024 | 2011-10-941-1 | 151 | not available in export | Denmark | 2011 | unknown | animal | 1696 | yes | not available in export | partial | Public PubMLST ST151 record |
| 201 | 55713 | 2011-10-942-1 | 151 | not available in export | Denmark | 2011 | unknown | animal | 1694 | yes | not available in export | partial | Public PubMLST ST151 record |
| 202 | 55715 | 2009-10-301 | 151 | not available in export | Denmark | 2009 | unknown | animal | 1699 | yes | not available in export | partial | Public PubMLST ST151 record |
| 203 | 55802 | E46 | 151 | not available in export | UK | 1991 | unknown | animal | 1700 | yes | not available in export | partial | Public PubMLST ST151 record |
| 204 | 55803 | E42 | 151 | not available in export | UK | 1991 | unknown | animal | 1700 | yes | not available in export | partial | Public PubMLST ST151 record |
| 205 | 55862 | 382 | 151 | not available in export | Switzerland | 1991 | unknown | animal | 1696 | yes | not available in export | partial | Public PubMLST ST151 record |
| 206 | 55867 | 350(DBDPCF01) | 151 | not available in export | Switzerland | 1991 | unknown | animal | 1698 | yes | not available in export | partial | Public PubMLST ST151 record |
| 207 | 55875 | A38 | 151 | not available in export | Denmark | 1991 | unknown | animal | 1698 | yes | not available in export | partial | Public PubMLST ST151 record |
| 208 | 56563 | T13/1/5 | 151 | not available in export | Australia | 2013 | unknown | animal | 1687 | yes | not available in export | partial | Public PubMLST ST151 record |
| 209 | 56612 | T13/1/32 | 151 | not available in export | Australia | 2013 | unknown | animal | 1702 | yes | not available in export | partial | Public PubMLST ST151 record |
| 210 | 56613 | V13/2/230 | 151 | not available in export | Australia | 2013 | unknown | animal | 1697 | yes | not available in export | partial | Public PubMLST ST151 record |
| 211 | 56693 | E30(DBDTFI01) | 151 | not available in export | UK | 1991 | unknown | animal | 1697 | yes | not available in export | partial | Public PubMLST ST151 record |
| 212 | 56702 | E22(DBDTFR01) | 151 | not available in export | UK | 1991 | unknown | animal | 1696 | yes | not available in export | partial | Public PubMLST ST151 record |
| 213 | 56709 | E8(DBDTFY01) | 151 | not available in export | Finland | 1991 | unknown | animal | 1701 | yes | not available in export | partial | Public PubMLST ST151 record |
| 214 | 56713 | E7(DBDTGC01) | 151 | not available in export | Finland | 1991 | unknown | animal | 1700 | yes | not available in export | partial | Public PubMLST ST151 record |
| 215 | 56797 | 95(DBDTJJ01) | 151 | not available in export | Ireland | 1991 | unknown | animal | 1697 | yes | not available in export | partial | Public PubMLST ST151 record |
| 216 | 56801 | 90(DBDTJN01) | 151 | not available in export | Ireland | 1991 | unknown | animal | 1697 | yes | not available in export | partial | Public PubMLST ST151 record |
| 217 | 56802 | 86 | 151 | not available in export | Ireland | 1991 | unknown | animal | 1695 | yes | not available in export | partial | Public PubMLST ST151 record |
| 218 | 56805 | 83(DBDTJR01) | 151 | not available in export | Ireland | 1991 | unknown | animal | 1697 | yes | not available in export | partial | Public PubMLST ST151 record |
| 219 | 56806 | 82(DBDTJS01) | 151 | not available in export | Ireland | 1991 | unknown | animal | 1696 | yes | not available in export | partial | Public PubMLST ST151 record |
| 220 | 56812 | 381 | 151 | not available in export | Switzerland | 1991 | unknown | animal | 1697 | yes | not available in export | partial | Public PubMLST ST151 record |
| 221 | 56819 | 354(DBDTKF01) | 151 | not available in export | Switzerland | 1991 | unknown | animal | 1697 | yes | not available in export | partial | Public PubMLST ST151 record |
| 222 | 56866 | A42 | 151 | not available in export | Denmark | 1991 | unknown | animal | 1697 | yes | not available in export | partial | Public PubMLST ST151 record |
| 223 | 56873 | A31 | 151 | not available in export | Denmark | 1991 | unknown | animal | 1698 | yes | not available in export | partial | Public PubMLST ST151 record |
| 224 | 56876 | A25 | 151 | not available in export | Denmark | 1991 | unknown | animal | 1698 | yes | not available in export | partial | Public PubMLST ST151 record |
| 225 | 56907 | 1657-LR | 151 | not available in export | USA | 1991 | unknown | animal | 1697 | yes | not available in export | partial | Public PubMLST ST151 record |
| 226 | 56919 | 93(DBDWYF01) | 151 | not available in export | Ireland | 1991 | unknown | animal | 1697 | yes | not available in export | partial | Public PubMLST ST151 record |
| 227 | 57618 | 31210331 | 151 | not available in export | Canada | 2007 | unknown | animal | 1699 | yes | not available in export | partial | Public PubMLST ST151 record |
| 228 | 57620 | 31000024 | 151 | not available in export | Canada | 2019 | unknown | animal | 1697 | yes | not available in export | partial | Public PubMLST ST151 record |
| 229 | 57621 | 51000033 | 151 | not available in export | Canada | 2019 | unknown | animal | 1698 | yes | not available in export | partial | Public PubMLST ST151 record |
| 230 | 57622 | 41000044 | 151 | not available in export | Canada | 2019 | unknown | animal | 1699 | yes | not available in export | partial | Public PubMLST ST151 record |
| 231 | 57625 | 21608872 | 151 | not available in export | Canada | 2007 | unknown | animal | 1696 | yes | not available in export | partial | Public PubMLST ST151 record |
| 232 | 57628 | 30500174 | 151 | not available in export | Canada | 2007 | unknown | animal | 1698 | yes | not available in export | partial | Public PubMLST ST151 record |
| 233 | 57629 | 30704176 | 151 | not available in export | Canada | 2007 | unknown | animal | 1696 | yes | not available in export | partial | Public PubMLST ST151 record |
| 234 | 57634 | 30600096 | 151 | not available in export | Canada | 2007 | unknown | animal | 1697 | yes | not available in export | partial | Public PubMLST ST151 record |
| 235 | 57635 | 41302682 | 151 | not available in export | Canada | 2007 | unknown | animal | 1697 | yes | not available in export | partial | Public PubMLST ST151 record |
| 236 | 57636 | 41704653 | 151 | not available in export | Canada | 2007 | unknown | animal | 1698 | yes | not available in export | partial | Public PubMLST ST151 record |
| 237 | 57641 | 30108394 | 151 | not available in export | Canada | 2007 | unknown | animal | 1696 | yes | not available in export | partial | Public PubMLST ST151 record |
| 238 | 57643 | 41103371 | 151 | not available in export | Canada | 2007 | unknown | animal | 1697 | yes | not available in export | partial | Public PubMLST ST151 record |
| 239 | 57649 | 10112441 | 151 | not available in export | Canada | 2008 | unknown | animal | 1697 | yes | not available in export | partial | Public PubMLST ST151 record |
| 240 | 57657 | SAU 0023 | 151 | not available in export | Canada | 2007 | unknown | animal | 1680 | yes | not available in export | partial | Public PubMLST ST151 record |
| 241 | 57658 | SAU 0027 | 151 | not available in export | Canada | 2007 | unknown | animal | 1650 | no | not available in export | partial | Public PubMLST ST151 record |
| 242 | 57669 | SAU 0362 | 151 | not available in export | Canada | 2007 | unknown | animal | 1686 | yes | not available in export | partial | Public PubMLST ST151 record |
| 243 | 57671 | SAU 0462 | 151 | not available in export | Canada | 2007 | unknown | animal | 1656 | yes | not available in export | partial | Public PubMLST ST151 record |
| 244 | 57673 | SAU 0540 | 151 | not available in export | Canada | 2007 | unknown | animal | 1662 | yes | not available in export | partial | Public PubMLST ST151 record |
| 245 | 57679 | SAU 0799 | 151 | not available in export | Canada | 2007 | unknown | animal | 1688 | yes | not available in export | partial | Public PubMLST ST151 record |
| 246 | 57685 | SAU 1021 | 151 | not available in export | Canada | 2007 | unknown | animal | 1653 | no | not available in export | partial | Public PubMLST ST151 record |
| 247 | 57686 | SAU 1052 | 151 | not available in export | Canada | 2008 | unknown | animal | 1682 | yes | not available in export | partial | Public PubMLST ST151 record |
| 248 | 57688 | SAU 1132a | 151 | not available in export | Canada | 2007 | unknown | animal | 1637 | no | not available in export | partial | Public PubMLST ST151 record |
| 249 | 57691 | SAU 1205 | 151 | not available in export | Canada | 2007 | unknown | animal | 1686 | yes | not available in export | partial | Public PubMLST ST151 record |
| 250 | 57693 | SAU 1212 | 151 | not available in export | Canada | 2008 | unknown | animal | 1646 | no | not available in export | partial | Public PubMLST ST151 record |
| 251 | 57694 | SAU 1300 | 151 | not available in export | Canada | 2007 | unknown | animal | 1687 | yes | not available in export | partial | Public PubMLST ST151 record |
| 252 | 57700 | SAU 1482 | 151 | not available in export | Canada | 2007 | unknown | animal | 1605 | no | not available in export | partial | Public PubMLST ST151 record |
| 253 | 57705 | SAU 1779 | 151 | not available in export | Canada | 2008 | unknown | animal | 1615 | no | not available in export | partial | Public PubMLST ST151 record |
| 254 | 57706 | SAU 1894 | 151 | not available in export | Canada | 2007 | unknown | animal | 1667 | yes | not available in export | partial | Public PubMLST ST151 record |
| 255 | 57708 | SAU 1997 | 151 | not available in export | Canada | 2007 | unknown | animal | 1603 | no | not available in export | partial | Public PubMLST ST151 record |
| 256 | 57711 | SAU 2025 | 151 | not available in export | Canada | 2007 | unknown | animal | 1687 | yes | not available in export | partial | Public PubMLST ST151 record |
| 257 | 57712 | SAU 2027 | 151 | not available in export | Canada | 2007 | unknown | animal | 1647 | no | not available in export | partial | Public PubMLST ST151 record |
| 258 | 57713 | SAU 2104 | 151 | not available in export | Canada | 2007 | unknown | animal | 1663 | yes | not available in export | partial | Public PubMLST ST151 record |
| 259 | 57718 | SAU 2526 | 151 | not available in export | Canada | 2008 | unknown | animal | 1605 | no | not available in export | partial | Public PubMLST ST151 record |
| 260 | 57720 | SAU 2811 | 151 | not available in export | Canada | 2008 | unknown | animal | 1676 | yes | not available in export | partial | Public PubMLST ST151 record |
| 261 | 57721 | SAU 2872 | 151 | not available in export | Canada | 2007 | unknown | animal | 1649 | no | not available in export | partial | Public PubMLST ST151 record |
| 262 | 57724 | SAU 2961 | 151 | not available in export | Canada | 2007 | unknown | animal | 1643 | no | not available in export | partial | Public PubMLST ST151 record |
| 263 | 57725 | SAU 2984 | 151 | not available in export | Canada | 2007 | unknown | animal | 1675 | yes | not available in export | partial | Public PubMLST ST151 record |
| 264 | 57727 | SAU 3057 | 151 | not available in export | Canada | 2008 | unknown | animal | 1667 | yes | not available in export | partial | Public PubMLST ST151 record |
| 265 | 57729 | SAU 3205 | 151 | not available in export | Canada | 2007 | unknown | animal | 1678 | yes | not available in export | partial | Public PubMLST ST151 record |
| 266 | 57731 | SAU 3457 | 151 | not available in export | Canada | 2007 | unknown | animal | 1683 | yes | not available in export | partial | Public PubMLST ST151 record |
| 267 | 57732 | SAU 3559 | 151 | not available in export | Canada | 2007 | unknown | animal | 1593 | no | not available in export | partial | Public PubMLST ST151 record |
| 268 | 57736 | SAU 4178 | 151 | not available in export | Canada | 2007 | unknown | animal | 1673 | yes | not available in export | partial | Public PubMLST ST151 record |
| 269 | 57740 | SAU 4415 | 151 | not available in export | Canada | 2007 | unknown | animal | 1575 | no | not available in export | partial | Public PubMLST ST151 record |
| 270 | 57741 | SAU 4506 | 151 | not available in export | Canada | 2007 | unknown | animal | 1693 | yes | not available in export | partial | Public PubMLST ST151 record |
| 271 | 57742 | SAU 4554 | 151 | not available in export | Canada | 2007 | unknown | animal | 1610 | no | not available in export | partial | Public PubMLST ST151 record |
| 272 | 57744 | SAU 5-SA | 151 | not available in export | Canada | 2007 | unknown | animal | 1659 | yes | not available in export | partial | Public PubMLST ST151 record |
| 273 | 57750 | SAU 6-SA | 151 | not available in export | Canada | 2007 | unknown | animal | 1689 | yes | not available in export | partial | Public PubMLST ST151 record |
| 274 | 57751 | SAU 6049 | 151 | not available in export | Canada | 2007 | unknown | animal | 1590 | no | not available in export | partial | Public PubMLST ST151 record |
| 275 | 57754 | SAU 7231 | 151 | not available in export | Canada | 2007 | unknown | animal | 1671 | yes | not available in export | partial | Public PubMLST ST151 record |
| 276 | 57755 | SAU 7404 | 151 | not available in export | Canada | 2007 | unknown | animal | 1583 | no | not available in export | partial | Public PubMLST ST151 record |
| 277 | 57756 | SAU 7526 | 151 | not available in export | Canada | 2007 | unknown | animal | 1604 | no | not available in export | partial | Public PubMLST ST151 record |
| 278 | 57759 | SAU 7829 | 151 | not available in export | Canada | 2007 | unknown | animal | 1673 | yes | not available in export | partial | Public PubMLST ST151 record |
| 279 | 57761 | SAU 8111 | 151 | not available in export | Canada | 2007 | unknown | animal | 1684 | yes | not available in export | partial | Public PubMLST ST151 record |
| 280 | 57768 | SAU 9255 | 151 | not available in export | Canada | 2007 | unknown | animal | 1694 | yes | not available in export | partial | Public PubMLST ST151 record |
| 281 | 57769 | SAU 9309 | 151 | not available in export | Canada | 2007 | unknown | animal | 1643 | no | not available in export | partial | Public PubMLST ST151 record |
| 282 | 58535 | Ani-GT-110 | 151 | not available in export | Belgium | 2016 | unknown | animal | 1699 | yes | not available in export | partial | Public PubMLST ST151 record |
| 283 | 58539 | Ani-GT-115 | 151 | not available in export | Belgium | 2016 | unknown | animal | 1696 | yes | not available in export | partial | Public PubMLST ST151 record |
| 284 | 58541 | Ani-GT-117 | 151 | not available in export | Belgium | 2016 | unknown | animal | 1699 | yes | not available in export | partial | Public PubMLST ST151 record |
| 285 | 59207 | 510579-22 | 151 | not available in export | Rwanda | 2021 | unknown | animal | 1697 | yes | not available in export | partial | Public PubMLST ST151 record |
| 286 | 60850 | 13_6_D | 151 | not available in export | Ireland | 2018 | mastitis | milk | 1690 | yes | not available in export | complete | Public PubMLST ST151 record |
| 287 | 60858 | 15_2_D | 151 | not available in export | Ireland | 2019 | mastitis | milk | 1685 | yes | not available in export | complete | Public PubMLST ST151 record |
| 288 | 60861 | 16_2_C | 151 | not available in export | Ireland | 2019 | mastitis | milk | 1697 | yes | not available in export | complete | Public PubMLST ST151 record |
| 289 | 60887 | 2_1_A | 151 | not available in export | Ireland | 2018 | mastitis | milk | 1694 | yes | not available in export | complete | Public PubMLST ST151 record |
| 290 | 60888 | 2_2_A | 151 | not available in export | Ireland | 2018 | mastitis | milk | 1692 | yes | not available in export | complete | Public PubMLST ST151 record |
| 291 | 60889 | 2_3_A | 151 | not available in export | Ireland | 2018 | mastitis | milk | 1688 | yes | not available in export | complete | Public PubMLST ST151 record |
| 292 | 60890 | 2_4_B | 151 | not available in export | Ireland | 2018 | mastitis | milk | 1697 | yes | not available in export | complete | Public PubMLST ST151 record |
| 293 | 60899 | 5_1_C | 151 | not available in export | Ireland | 2019 | mastitis | milk | 1694 | yes | not available in export | complete | Public PubMLST ST151 record |
| 294 | 60900 | 5_2_BD | 151 | not available in export | Ireland | 2019 | mastitis | milk | 1695 | yes | not available in export | complete | Public PubMLST ST151 record |
| 295 | 60902 | 5_3_C | 151 | not available in export | Ireland | 2019 | mastitis | milk | 1694 | yes | not available in export | complete | Public PubMLST ST151 record |
| 296 | 60904 | 5_5_B | 151 | not available in export | Ireland | 2019 | mastitis | milk | 1695 | yes | not available in export | complete | Public PubMLST ST151 record |
| 297 | 60908 | 5_9_C | 151 | not available in export | Ireland | 2019 | mastitis | milk | 1695 | yes | not available in export | complete | Public PubMLST ST151 record |
| 298 | 60909 | 6_1_D | 151 | not available in export | Ireland | 2019 | mastitis | milk | 1697 | yes | not available in export | complete | Public PubMLST ST151 record |
| 299 | 60910 | 6_2_D | 151 | not available in export | Ireland | 2019 | mastitis | milk | 1690 | yes | not available in export | complete | Public PubMLST ST151 record |
| 300 | 60911 | 7_1_BD | 151 | not available in export | Ireland | 2019 | mastitis | milk | 1692 | yes | not available in export | complete | Public PubMLST ST151 record |
| 301 | 60912 | 7_2_D | 151 | not available in export | Ireland | 2019 | mastitis | milk | 1691 | yes | not available in export | complete | Public PubMLST ST151 record |
| 302 | 60913 | 7_3_B | 151 | not available in export | Ireland | 2019 | mastitis | milk | 1692 | yes | not available in export | complete | Public PubMLST ST151 record |
| 303 | 60914 | 7_4_C | 151 | not available in export | Ireland | 2019 | mastitis | milk | 1692 | yes | not available in export | complete | Public PubMLST ST151 record |
| 304 | 60915 | 7_5_B | 151 | not available in export | Ireland | 2019 | mastitis | milk | 1692 | yes | not available in export | complete | Public PubMLST ST151 record |
| 305 | 60916 | 8_1_AC | 151 | not available in export | Ireland | 2019 | mastitis | milk | 1687 | yes | not available in export | complete | Public PubMLST ST151 record |
| 306 | 60917 | 8_10_B | 151 | not available in export | Ireland | 2019 | mastitis | milk | 1689 | yes | not available in export | complete | Public PubMLST ST151 record |
| 307 | 60918 | 8_11_BD | 151 | not available in export | Ireland | 2019 | mastitis | milk | 1688 | yes | not available in export | complete | Public PubMLST ST151 record |
| 308 | 60919 | 8_12_CD | 151 | not available in export | Ireland | 2019 | mastitis | milk | 1688 | yes | not available in export | complete | Public PubMLST ST151 record |
| 309 | 60920 | 8_13_CD | 151 | not available in export | Ireland | 2019 | mastitis | milk | 1690 | yes | not available in export | complete | Public PubMLST ST151 record |
| 310 | 60921 | 8_2_B | 151 | not available in export | Ireland | 2019 | mastitis | milk | 1688 | yes | not available in export | complete | Public PubMLST ST151 record |
| 311 | 60923 | 8_4_A | 151 | not available in export | Ireland | 2019 | mastitis | milk | 1687 | yes | not available in export | complete | Public PubMLST ST151 record |
| 312 | 60924 | 8_4_D | 151 | not available in export | Ireland | 2019 | mastitis | milk | 1690 | yes | not available in export | complete | Public PubMLST ST151 record |
| 313 | 60927 | 8_8_A | 151 | not available in export | Ireland | 2019 | mastitis | milk | 1690 | yes | not available in export | complete | Public PubMLST ST151 record |
| 314 | not applicable | SA_KSRVI_2025_1 | 151 | closest cgST 32654 | Kazakhstan | 2025 | clinical mastitis | bovine milk | 1654/1716 matched loci reported for closest profile comparison | query isolate from present study | 62 alleles to closest cgST profile (cgST-32654) | complete | Kazakhstan query isolate from present study; not present in the uploaded PubMLST export files |
