# Supplementary material for: Single-isolate genomic characterization and cgMLST allelic comparison of a bovine mastitis-associated Staphylococcus aureus ST151 isolate from Kazakhstan
Source: Front Vet Sci. 2026 Jul 30;13:1881180. doi: 10.3389/fvets.2026.1881180 (PMC13467824; doi:10.3389/fvets.2026.1881180)
Supplement: Supplementary file 2 [file Table_2.docx]

**Supplementary Table S2.**

**Gene-level summary of virulence-associated loci detected by VirulenceFinder 2.0 in the genome of the sequenced Staphylococcus aureus isolate.**

The table includes gene name, identity, query/template length, coverage, functional category, predicted function, accession number, and final interpretation. Genes seg, sei, sem, sen, seo, and seu were interpreted as egc-associated enterotoxin-like genes rather than as evidence of classical enterotoxin activity. Detection of these genes was not considered evidence of toxin expression, toxin production, or food poisoning risk.

| **Gene** | **Tool/database** | **Identity (%)** | **Query / template length** | **Coverage (%)** | **Functional category** | **Predicted function** | **Accession number** | **Final interpretation** |
| --- | --- | --- | --- | --- | --- | --- | --- | --- |
| *aur* | VirulenceFinder 2.0 | 100.00 | 1530 / 1530 | 100.00 | Exoenzyme | Aureolysin | AJ938182.1 | Virulence-associated exoenzyme locus; expression and functional activity were not tested |
| *splA* | VirulenceFinder 2.0 | 97.74 | 707 / 708 | 99.86 | Exoenzyme / serine protease | Serine protease SplA | CP006706.1; FR821779.1 | Virulence-associated protease locus; expression and functional activity were not tested |
| *splB* | VirulenceFinder 2.0 | 97.65 | 723 / 723 | 100.00 | Exoenzyme / serine protease | Serine protease SplB | FR821779.1 | Virulence-associated protease locus; expression and functional activity were not tested |
| *hlgA* | VirulenceFinder 2.0 | 99.89 | 930 / 930 | 100.00 | Hemolysin | Gamma-hemolysin chain II precursor | AJ938182.1 | Virulence-associated hemolysin locus; cytotoxic activity was not experimentally tested |
| *hlgB* | VirulenceFinder 2.0 | 99.80 | 977 / 977 | 100.00 | Hemolysin | Gamma-hemolysin component B precursor | AJ938182.1 | Virulence-associated hemolysin locus; cytotoxic activity was not experimentally tested |
| *hlgC* | VirulenceFinder 2.0 | 100.00 | 948 / 948 | 100.00 | Hemolysin | Gamma-hemolysin component C | AJ938182.1 | Virulence-associated hemolysin locus; cytotoxic activity was not experimentally tested |
| *lukD* | VirulenceFinder 2.0 | 100.00 | 983 / 983 | 100.00 | Leukocidin | Leukocidin D component | AJ938182.1 | Virulence-associated leukocidin locus; leukotoxic activity was not experimentally tested |
| *lukE* | VirulenceFinder 2.0 | 100.00 | 936 / 936 | 100.00 | Leukocidin | Leukocidin E component | AJ938182.1 | Virulence-associated leukocidin locus; leukotoxic activity was not experimentally tested |
| *seg* | VirulenceFinder 2.0 | 99.87 | 776 / 776 | 100.00 | egc-associated enterotoxin-like gene | Enterotoxin G | AJ938182.1 | Interpreted as egc-associated enterotoxin-like gene; toxin expression/production was not assessed |
| *sei* | VirulenceFinder 2.0 | 100.00 | 729 / 729 | 100.00 | egc-associated enterotoxin-like gene | Enterotoxin I | AJ938182.1 | Interpreted as egc-associated enterotoxin-like gene; toxin expression/production was not assessed |
| *sem* | VirulenceFinder 2.0 | 99.86 | 721 / 721 | 100.00 | egc-associated enterotoxin-like gene | Enterotoxin M | AJ938182.1 | Interpreted as egc-associated enterotoxin-like gene; toxin expression/production was not assessed |
| *sen* | VirulenceFinder 2.0 | 97.68 | 777 / 777 | 100.00 | egc-associated enterotoxin-like gene | Enterotoxin N | BA000018.3 | Interpreted as egc-associated enterotoxin-like gene; toxin expression/production was not assessed |
| *seo* | VirulenceFinder 2.0 | 100.00 | 780 / 780 | 100.00 | egc-associated enterotoxin-like gene | Enterotoxin O | AJ938182.1 | Interpreted as egc-associated enterotoxin-like gene; toxin expression/production was not assessed |
| *seu* | VirulenceFinder 2.0 | 97.71 | 786 / 786 | 100.00 | egc-associated enterotoxin-like gene | Enterotoxin U | CP002388.1; CP006706.1 | Interpreted as egc-associated enterotoxin-like gene; toxin expression/production was not assessed |
