# Supplementary material for: Single-isolate genomic characterization and cgMLST allelic comparison of a bovine mastitis-associated Staphylococcus aureus ST151 isolate from Kazakhstan
Source: Front Vet Sci. 2026 Jul 30;13:1881180. doi: 10.3389/fvets.2026.1881180 (PMC13467824; doi:10.3389/fvets.2026.1881180)
Supplement: Supplementary file 3 [file Table_3.docx]

**Supplementary Table S3**

**Gene-level summary of antimicrobial resistance-associated loci detected by CARD RGI in the genome of the sequenced Staphylococcus aureus isolate.**

The table includes CARD RGI criteria, identity, reference sequence coverage, predicted drug class, resistance mechanism, AMR gene family, and cautious interpretation. CARD RGI hits were interpreted together with ResFinder and PointFinder results. ResFinder did not detect acquired antimicrobial resistance genes, and PointFinder did not identify known resistance-associated point mutations; therefore, these loci were not interpreted alone as evidence of categorical antimicrobial resistance or multidrug resistance.

| **Gene / ARO term** | **Tool / database** | **RGI criteria** | **Identity (%)** | **Reference length (%)** | **Drug class / antibiotic** | **Resistance mechanism** | **AMR gene family** | **Final interpretation** |
| --- | --- | --- | --- | --- | --- | --- | --- | --- |
| *Staphylococcus aureus norA* | CARD RGI | Strict | 97.16 | 100.00 | Fluoroquinolone antibiotics; enoxacin, ciprofloxacin, norfloxacin, ofloxacin | Antibiotic efflux | Major facilitator superfamily (MFS) antibiotic efflux pump | Efflux-associated locus; not interpreted alone as categorical fluoroquinolone resistance |
| *mgrA* | CARD RGI | Perfect | 100.00 | 100.00 | Fluoroquinolone, cephalosporin, penicillin beta-lactam, tetracycline, peptide, moenomycin antibiotics; disinfectants/antiseptics | Antibiotic efflux | ABC and MFS antibiotic efflux pump-associated regulator | Regulatory/efflux-associated locus; not interpreted alone as multidrug resistance |
| *mepR* | CARD RGI | Perfect | 100.00 | 100.00 | Glycylcycline and tetracycline antibiotics; tigecycline | Antibiotic efflux | MATE transporter-associated regulator | Efflux-regulatory locus; phenotypic impact not confirmed |
| *norC* | CARD RGI | Perfect | 100.00 | 100.00 | Fluoroquinolone antibiotics and disinfectants/antiseptics; ciprofloxacin, acriflavine, norfloxacin | Antibiotic efflux | MFS antibiotic efflux pump | Efflux-associated locus; not interpreted alone as categorical resistance |
| *sdrM* | CARD RGI | Strict | 98.88 | 100.00 | Fluoroquinolone antibiotics and disinfectants/antiseptics; norfloxacin | Antibiotic efflux | MFS antibiotic efflux pump | Efflux-associated locus; not interpreted alone as multidrug resistance |
| *sepA* | CARD RGI | Strict | 95.48 | 98.73 | Disinfecting agents and antiseptics; acriflavine | Antibiotic efflux | Small multidrug resistance (SMR) antibiotic efflux pump | Efflux-associated locus; not interpreted as clinical antimicrobial resistance |
| *Staphylococcus aureus LmrS* | CARD RGI | Strict | 97.29 | 100.00 | Macrolide, aminoglycoside, oxazolidinone, diaminopyrimidine, and phenicol antibiotics | Antibiotic efflux | MFS antibiotic efflux pump | Efflux-associated locus; requires phenotypic confirmation |
| *kdpD* | CARD RGI | Strict | 98.76 | 100.00 | Aminoglycoside antibiotic; kanamycin A | Antibiotic efflux | kdpDE regulatory system | Regulatory locus; not interpreted alone as aminoglycoside resistance |
| *vanT* gene in *vanG* cluster | CARD RGI | Strict | 33.33 | 53.65 | Glycopeptide antibiotic; vancomycin | Antibiotic target alteration | Glycopeptide resistance gene cluster; *vanT* | Low-identity/partial hit; not interpreted as vancomycin resistance |
| *norC*-like hit | CARD RGI | Strict | 70.20 | 100.22 | Fluoroquinolone antibiotics and disinfectants/antiseptics; ciprofloxacin, acriflavine, norfloxacin | Antibiotic efflux | MFS antibiotic efflux pump | Additional lower-identity *norC*-like hit; interpreted cautiously |
| *arlR* | CARD RGI | Perfect | 100.00 | 100.00 | Fluoroquinolone antibiotics and disinfectants/antiseptics; ciprofloxacin, acriflavine, norfloxacin | Antibiotic efflux | MFS antibiotic efflux pump-associated regulatory locus | Regulatory locus; biological impact requires functional confirmation |
| *Staphylococcus aureus GlpT with mutation conferring resistance to fosfomycin* | CARD RGI | Strict | 99.12 | 100.00 | Phosphonic acid antibiotic; fosfomycin | Antibiotic target alteration | Antibiotic-resistant GlpT | CARD variant-model hit; interpreted cautiously because ResFinder/PointFinder did not predict fosfomycin resistance |
| *Staphylococcus aureus murA with mutation conferring resistance to fosfomycin* | CARD RGI | Strict | 99.29 | 100.00 | Phosphonic acid antibiotic; fosfomycin | Antibiotic target alteration | Antibiotic-resistant MurA transferase | CARD variant-model hit; interpreted cautiously because ResFinder/PointFinder did not predict fosfomycin resistance |
