# Supplementary material for: Single-isolate genomic characterization and cgMLST allelic comparison of a bovine mastitis-associated Staphylococcus aureus ST151 isolate from Kazakhstan
Source: Front Vet Sci. 2026 Jul 30;13:1881180. doi: 10.3389/fvets.2026.1881180 (PMC13467824; doi:10.3389/fvets.2026.1881180)
Supplement: Supplementary file 1 [file Image_1.pdf]

Supplementary Figure S1.
Representative disk diffusion plates used for supportive phenotypic antimicrobial susceptibility screening of the sequenced *Staphylococcus aureus* isolate.

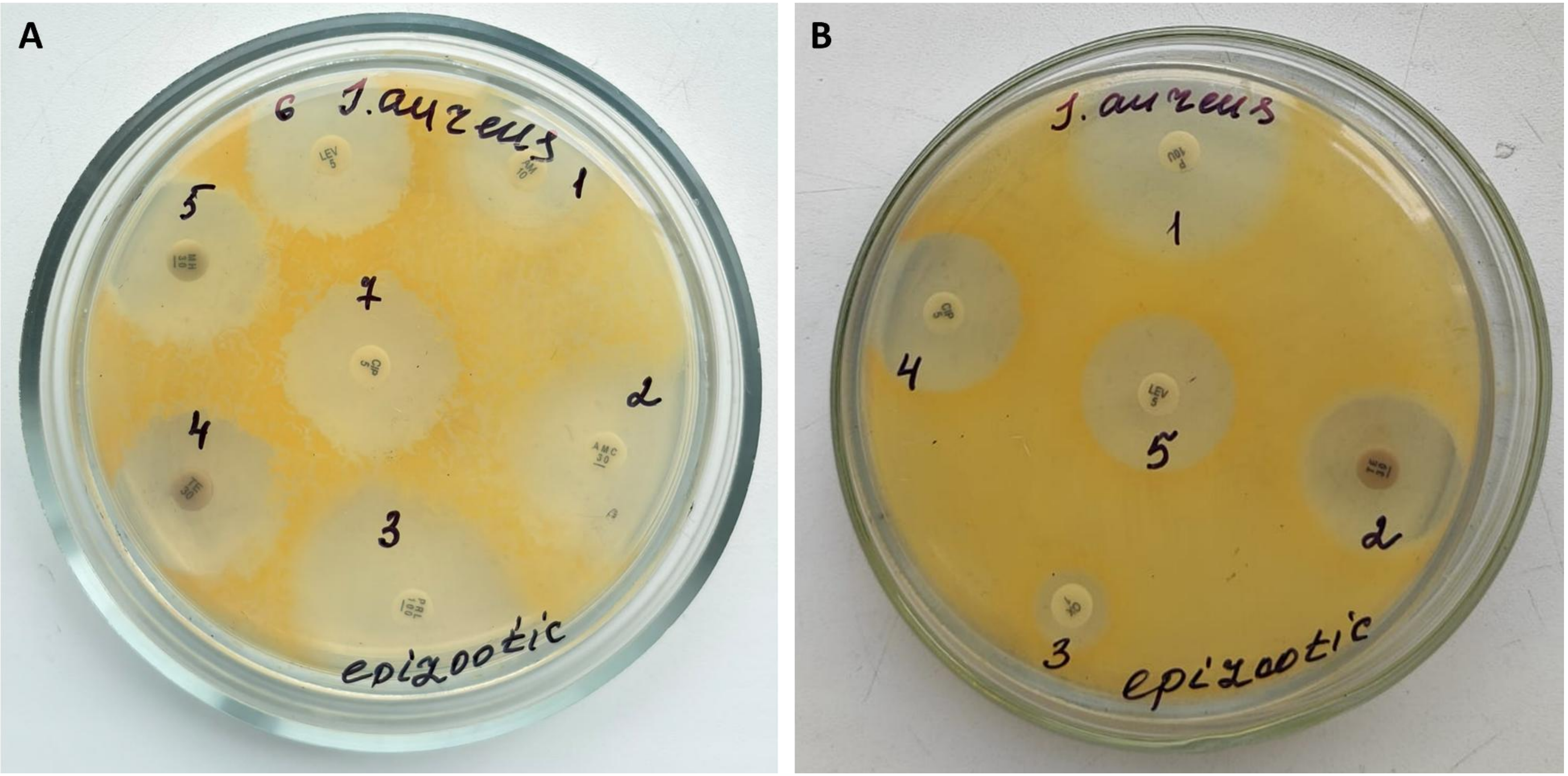

(A) Representative plate with seven antibiotic disks: 1, ampicillin (AM); 2, amoxicillin/clavulanic acid (AMC); 3, piperacillin (PRL); 4, tetracycline (TE); 5, minocycline (MH); 6, levofloxacin (LEV); and 7, ciprofloxacin (CIP).

(B) Representative plate with five antibiotic disks: 1, penicillin (P); 2, tetracycline (TE); 3, oxacillin (OX-1); 4, ciprofloxacin (CIP); and 5, levofloxacin (LEV).
